# The Roles of Cholesterol and Its Metabolites in Normal and Malignant Hematopoiesis

**DOI:** 10.3389/fendo.2019.00204

**Published:** 2019-04-02

**Authors:** Hideyuki Oguro

**Affiliations:** Cellular Engineering, The Jackson Laboratory for Genomic Medicine, Farmington, CT, United States

**Keywords:** cholesterol, oxysterols, steroids, hematopoietic stem cells, hematopoiesis, hematologic malignancies

## Abstract

Hematopoiesis is sustained throughout life by hematopoietic stem cells (HSCs) that are capable of self-renewal and differentiation into hematopoietic progenitor cells (HPCs). There is accumulating evidence that cholesterol homeostasis is an important factor in the regulation of hematopoiesis. Increased cholesterol levels are known to promote proliferation and mobilization of HSCs, while hypercholesterolemia is associated with expansion of myeloid cells in the peripheral blood and links hematopoiesis with cardiovascular disease. Cholesterol is a precursor to steroid hormones, oxysterols, and bile acids. Among steroid hormones, 17β-estradiol (E2) induces HSC division and E2-estrogen receptor α (ERα) signaling causes sexual dimorphism of HSC division rate. Oxysterols are oxygenated derivatives of cholesterol and key substrates for bile acid synthesis and are considered to be bioactive lipids, and recent studies have begun to reveal their important roles in the hematopoietic and immune systems. 27-Hydroxycholesterol (27HC) acts as an endogenous selective estrogen receptor modulator and induces ERα-dependent HSC mobilization and extramedullary hematopoiesis. 7α,25-dihydroxycholesterol (7α,25HC) acts as a ligand for Epstein-Barr virus-induced gene 2 (EBI2) and directs migration of B cells in the spleen during the adaptive immune response. Bile acids serve as chemical chaperones and alleviate endoplasmic reticulum stress in HSCs. Cholesterol metabolism is dysregulated in hematologic malignancies, and statins, which inhibit *de novo* cholesterol synthesis, have cytotoxic effects in malignant hematopoietic cells. In this review, recent advances in our understanding of the roles of cholesterol and its metabolites as signaling molecules in the regulation of hematopoiesis and hematologic malignancies are summarized.

## Introduction

**Figure 1:**
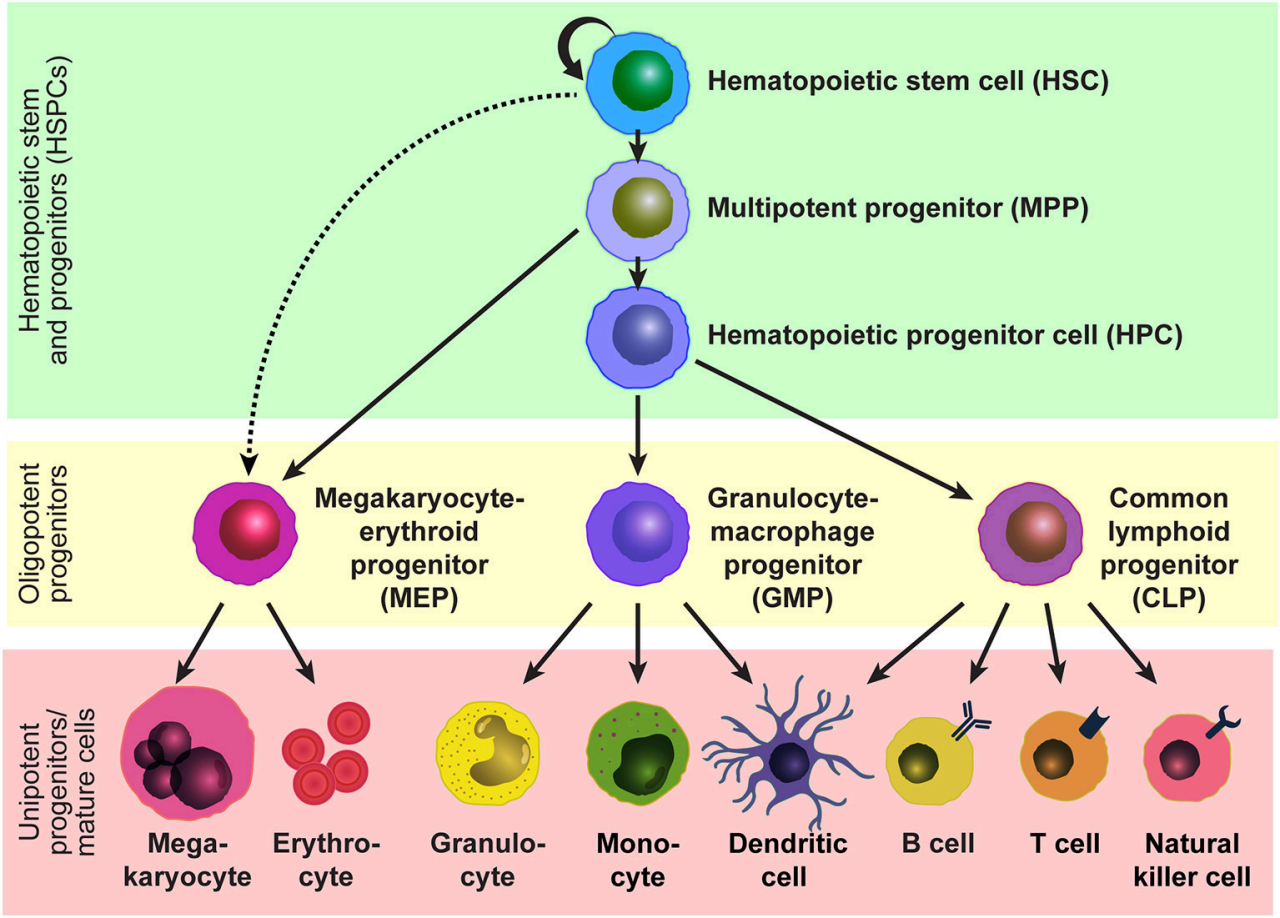
Model of the hematopoietic hierarchy. A hierarchical structure of the adult hematopoietic system based on studies by us and others using mice. HSCs reside at the top of the hierarchy and give rise to MPPs and potentially MEPs. MPPs generate HPCs and MEPs, and HPCs generate GMPs and CLPs. Mature cells are developed from these oligopotent progenitors, MEPs, GMPs, and CMPs, through intermediate progenitors. Some lineage relationships are under debate.

Hematopoietic stem cells (HSCs) sustain blood production throughout life and are the functional units of bone marrow transplantation. HSCs are capable of self-renewal to maintain their pool while producing all mature blood cells through differentiation into multipotent progenitors (MPPs) and subsequent hematopoietic progenitor cells (HPCs) with limited differentiation potentials (Figure 1). In adult mice, all HSCs and MPPs fall within the Lineage marker^−^Sca-1^+^c-Kit^+^ (LSK) fraction (1–3), which is a heterogeneous population that contains a mixture of hematopoietic stem and progenitor cells (HSPCs), including HSCs, MPPs, and HPCs. HSCs can be further purified by selecting the CD150^+^CD48^−/low^ subset (4, 5) or CD34^−/low^Flt3^−^ subset (6, 7) of LSK cells. The HSC population is functionally heterogenous in terms of cell-cycle kinetics, self-renewal capacity, and differentiation potential, and the heterogeneity can be distinguished by additional markers such as CD229 (5) and von Willebrand factor (8). Human HSPCs can be marked by Lineage marker^−^CD38^−^CD34^+^ and the HSC population can be further refined by marking the CD45RA^−^CD49f^+^ subset of Lineage marker^−^CD38^−^CD34^+^ cells (9). HSCs reside in specialized niches which are local tissue microenvironments that support HSC behavior and regulate their function, such as self-renewal, differentiation, and localization, by producing factors that act directly on HSCs (10). In adults, HSCs are quiescent and localize primarily in the bone marrow, and their number is tightly regulated under steady-state conditions, comprising < 0.01% of bone marrow cells in mice. In response to acute hematopoietic demands such as blood loss, myeloablation, infection, or pregnancy, HSCs change two aspects of their steady-state behaviors in order to increase production of necessary hematopoietic cells (11–14). First, quiescent HSCs re-enter the cell cycle to proliferate or differentiate through symmetric or asymmetric cell divisions, and second, they mobilize from the bone marrow to extramedullary tissues, such as the spleen, to expand the physical space for hematopoiesis.

**Figure 2:**
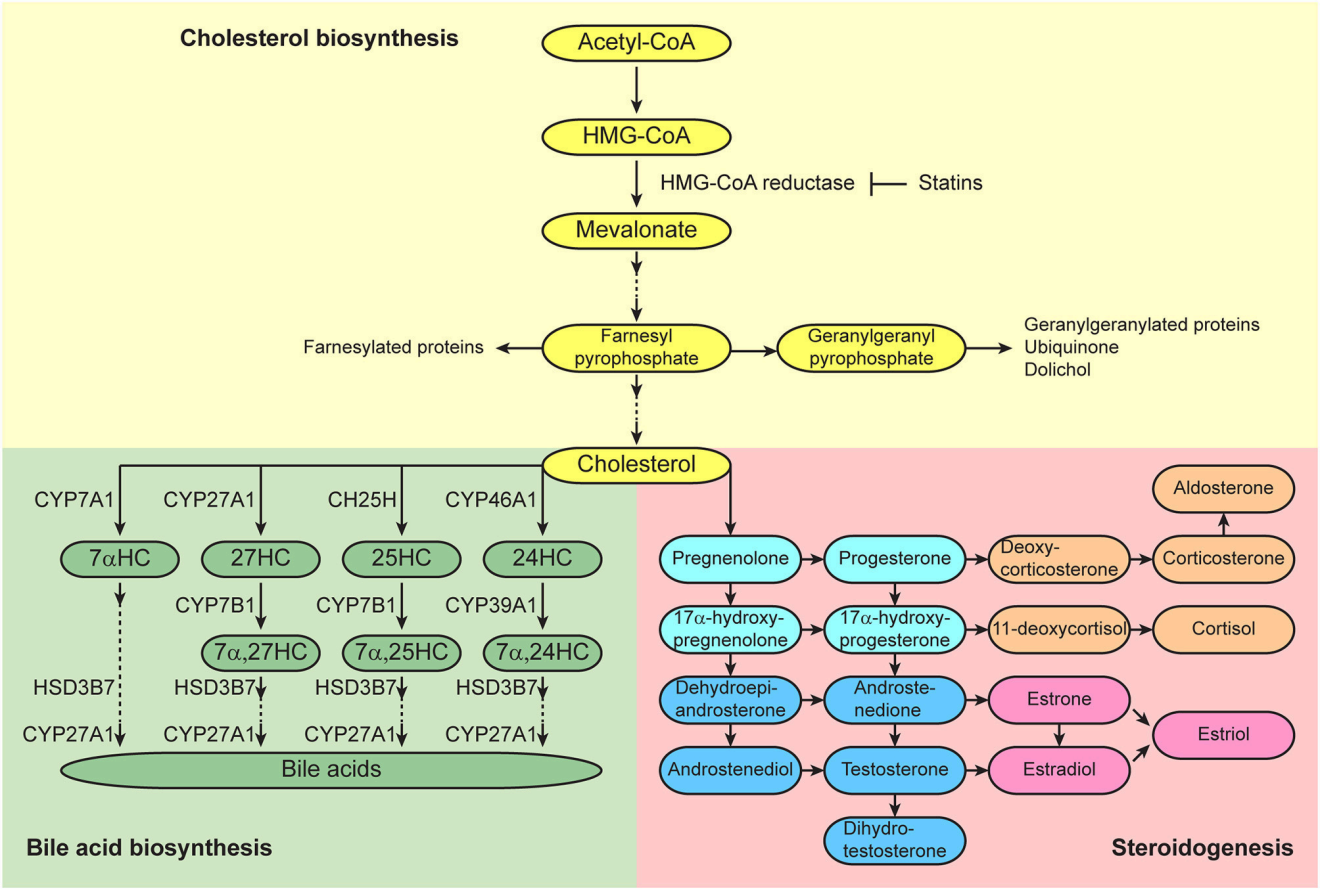
Cholesterol synthetic/metabolic pathways. Pathways of cholesterol biosynthesis (top), bile acid biosynthesis (bottom left), and steroidogenesis (bottom right) are presented. Enzymes that catalyze the conversions are shown adjacent to the arrows.

In addition to the regulation of HSC behaviors by short-range factors, such as cytokines, cell-surface proteins, extracellular matrix components, oxygen tension, and ion levels, that are generated in their niches, HSCs are also regulated by long-range systemic signals, such as circulating cytokines, hormones, lipids, and vitamins. Cholesterol is found in the bloodstream and within cells, and is an essential structural component of mammalian plasma membranes and is essential to maintain both membrane structural integrity and to modulate membrane fluidity (15). Cholesterol also serves as a precursor for the biosynthesis of steroid hormones, oxysterols, and bile acids (Figure 2) (16). These cholesterol metabolites have important biological roles as signal transducers and chemical chaperones, and there is accumulating evidence that these metabolites act as systemic signals that regulate normal and malignant hematopoiesis. This review discusses recent advances in understanding the roles of cholesterol and its metabolites in the regulation of hematopoiesis and hematologic malignancies.

## Cholesterol Levels and Hematopoiesis

In the bloodstream, cholesterol is transported within lipoprotein particles, which are organized by apolipoproteins that can be recognized and bound by specific receptors on cell membranes. There are several types of lipoproteins, such as high-density lipoprotein (HDL), low-density lipoprotein (LDL), intermediate-density lipoprotein (IDL), very-low-density lipoprotein (VLDL), and chylomicrons, in order of higher density to lower density. LDL is the major cholesterol carrier in the blood and is recognized by the LDL receptor (LDLR) in peripheral tissues. LDL is the atherogenic lipoprotein, and increased LDL levels promote cholesterol accumulation. Macrophages accumulate oxidized LDL and give rise to foam cells, and contribute to atherosclerotic plaque formation. HDL opposes this process and reduces inflammation. HDL is involved in reverse cholesterol transport, in which HDL serves to shuttle cholesterol from peripheral tissues to the liver where cholesterol is eventually converted into bile acids.

### Hypercholesterolemia Induces Proliferation and Mobilization of Mouse Hematopoietic Stem and Progenitor Cells

Several studies have shown a strong correlation between plasma cholesterol levels and the mobilized HSC number in the peripheral blood using mouse models. Gomes et al. showed that mice fed a high-fat/high-cholesterol (HFHC) diet for 30 days displayed thrombocytosis, lymphocytosis, and an increase in the number of HSPCs mobilized into the peripheral blood, while the HSPC number in the bone marrow decreased (17). They also found that the HFHC diet induced increased plasma levels of C-X-C motif chemokine ligand 12/ stromal cell-derived factor 1 (CXCL12/SDF1), a chemokine which is chemotactic for HSCs that express its receptor C-X-C motif chemokine receptor 4 (CXCR4) (18). Apolipoprotein E (APOE) is a key component in cholesterol metabolism and *Apoe*-deficient mice cause hypercholesterolemia (19, 20). *Apoe*-deficient mice fed an HFHC diet developed monocytosis (21–23), and Murphy et al. also reported neutrophilia associated with the proliferation and expansion of HSPCs in the bone marrow (23). Interestingly, APOE was expressed on the surface of HSPCs and acted cell autonomously to control HSPC proliferation, monocytosis, neutrophilia, and monocyte accumulation in atherosclerotic lesions, as revealed by transplantation of *Apoe*-deficient bone marrow cells. LDLR deficiency causes impaired LDL clearance, resulting in high plasma LDL-cholesterol levels and causing familial hypercholesterolemia. LDL receptor-deficient (*Ldlr*^−/−^) mice fed an HFHC diet displayed hypercholesterolemia associated with increased HSPCs in both bone marrow and peripheral blood, and increased monocytes and granulocytes in the peripheral blood (24, 25). In addition to their mobilization, more HSPCs in the bone marrow incorporated the DNA synthesis marker 5-bromo-2′-deoxyuridine (BrdU) in *Ldlr*^−/−^ mice on an HFHC diet as compared to in *Ldlr*^−/−^ mice on a normal diet, indicating that hypercholesterolemia promoted HSPC proliferation. In contrast, infusion with reconstituted HDL reduced the frequency and proliferation rate of HSPCs in the bone marrow, highlighting the opposing effects of LDL and HDL on HSPC proliferation. Scavenger receptor type BI (SR-BI, encoded by *Scarb1* gene) is a HDL receptor, and *Scarb1*^−/−^ mice showed increased plasma total cholesterol levels with unchanged plasma concentration of apoA-I, the major protein in HDL (26). Gao et al. reported that *Scarb1*^−/−^ mice fed an HFHC diet showed significantly increased the number of HSPCs in the bone marrow, spleen, and peripheral blood, as well as the proliferation of HSPCs as compared to wild-type mice fed an HFHC diet (27). Interestingly, HSPCs in *Scarb1*^−/−^ mice fed an HFHC diet displayed increased levels of reactive oxygen species (ROS). Elevation of ROS levels hinders HSC quiescence and self-renewal, and accelerates HSC exhaustion (28). Injection of ROS inhibitor N-acetylcysteine attenuated HSPC expansion and leukocytosis in *Scarb1*^−/−^ mice fed an HFHC diet, suggesting a correlation between ROS levels and HSPC proliferation. Tie et al. also reported that *Apoe*-deficiency increased the number and ROS levels of HSPCs, and these were further increased by the HFHC diet (29). They also observed shorter telomere length in HSPCs in *Apoe*^−/−^ mice as compared to wild-type mice, suggesting accelerated aging of HSPCs, and this phenotype was reversed by treating *Apoe*^−/−^ mice with N-acetylcysteine. These studies clearly indicate increased systemic cholesterol levels promote proliferation and mobilization of HSPCs.

### Cholesterol Efflux Pathways Regulate Proliferation and Mobilization of Mouse Hematopoietic Stem and Progenitor Cells

ABCA1 and ABCG1, adenosine triphosphate-binding cassette transporters, play a key role in promoting active cellular cholesterol efflux (30). Yvan-Charvet et al. reported that *Ldlr*^−/−^ mice on an HFHC diet that were transplanted with *Abca1*^−/−^*Abcg1*^−/−^ bone marrow cells showed accelerated atherosclerosis and extensive infiltration of myocardium and spleen with macrophage foam cells as compared to transplantation with wild-type bone marrow cells (31). The same group subsequently reported that *Abca1*^−/−^*Abcg1*^−/−^ mice on a normal diet displayed five-fold increase of HSPCs (including the CD34^+^CD150^+^Flt3^−^ highly-pure HSC population) in the bone marrow, as well as the increase in the S/G2/M fraction in HSPCs (32). The overall BrdU incorporation of *Abca1*^−/−^*Abcg1*^−/−^ bone marrow cells was increased *in vitro*, whereas when wild-type bone marrow cells were mixed with *Abca1*^−/−^*Abcg1*^−/−^ bone marrow cells, the overall BrdU incorporation of wild-type bone marrow cells was not increased, suggesting that HSPC proliferation in *Abca1*^−/−^*Abcg1*^−/−^ mice was caused by cell autonomous effects. Interestingly, in their next report, they showed that *Abca1*^−/−^*Abcg1*^−/−^ mice also displayed an increase in HSPCs in the peripheral blood, spleen, and liver, indicating HSPC mobilization and extramedullary hematopoiesis (33). In this study, they performed a competitive bone marrow transplantation experiment by transplanting a mixture of equal numbers of bone marrow cells from wild-type and *Abca1*^−/−^ *Abcg1*^−/−^ mice into wild-type recipient mice, and found that HSPC mobilization of both the *Abca1*^−/−^ *Abcg1*^−/−^ and wild-type donor cells was induced, suggesting that there is a cell-extrinsic factor that induces HSPC mobilization of wild-type donor cells from *Abca1*^−/−^*Abcg1*^−/−^ donor cells. Plasma levels of granulocyte colony-stimulating factor (G-CSF) were significantly increased in recipients of *Abca1*^−/−^*Abcg1*^−/−^ bone marrow cells and the mobilization of *Abca1*^−/−^*Abcg1*^−/−^ HSPCs was reduced by injection of G-CSF-neutralizing antibody. Interleukin-17 (IL-17) is a potent inducer of G-CSF (34), and the production of IL-17 can be mediated by the secretion of interleukin-23 (IL-23) from splenic phagocytic macrophages and dendritic cells (35). In the recipients of *Abca1*^−/−^*Abcg1*^−/−^ bone marrow cells, plasma G-CSF levels and colony-forming HSPC numbers in the blood were normalized by an IL-17-blocking antibody, and plasma levels of IL-17 and G-CSF, as well as colony-forming HSPCs in the blood, were reduced by administration of IL-23 receptor-neutralizing antibody. Both myeloid cell (including macrophages) -specific and dendritic-cell specific deletion of *Abca1* and *Abcg1* using *lysM-cre*; *Abca1*^*fl*/*fl*^; *Abcg1*^*fl*/*fl*^ mice, and *CD11c-cre*; *Abca1*^*fl*/*fl*^; *Abcg1*^*fl*/*fl*^ mice, respectively, exhibited increased levels of splenic IL-23, plasma IL-17 and G-CSF, and colony-forming HSPCs in the blood, suggesting that IL-23/IL-17/G-CSF signaling is associated with enhanced HSPC mobilization in *Abca1*^−/−^*Abcg1*^−/−^ mice. They further reported that CXCL12 levels and the number of N-Cadherin^+^ osteoblasts, one of the CXCL12-expressing cell populations in the bone marrow (36), were decreased in the bone marrow of *Abca1*^−/−^*Abcg1*^−/−^ mice, an effect that might be caused by depletion of bone marrow macrophage populations due to an enhanced IL-23/IL-17/G-CSF signaling axis. Thus, this study supports a step-wise mechanism by which increased intracellular cholesterol levels lead to mobilization of HSCs: (1) increased cholesterol initially promotes secretion of pro-inflammatory cytokines from immune cells, (2) this increases production of G-CSF by bone marrow stromal cells, (3) reducing osteoblast number and osteoblast production of CXCL12, a chemokine which attracts HSCs, and (4) leads to HSC mobilization into the bloodstream. They subsequently reported that *Ldlr*^−/−^ recipient mice that received *lysM-cre*; *Abca1*^*fl*/*fl*^; *Abcg1*^*fl*/*fl*^ bone marrow cells and were fed an HFHC diet developed atherosclerosis associated with monocytosis and neutrophilia (37). The authors demonstrated a cell-extrinsic mechanism in which the expression of macrophage colony-stimulating factor (M-CSF) and G-CSF were increased in the spleen, and this might cause monocyte and neutrophil production in the bone marrow.

### Cholesterol Levels and Human Hematopoiesis

Cholesterol homeostasis also affects human hematopoiesis. Crysandt et al. performed a retrospective analysis of a variety of clinical parameters in 83 patients following high-dose cyclophosphamide and G-CSF treatment and found that patients with hypercholesterolemia showed a substantially higher number of harvested CD34^+^ HSPCs in the peripheral blood as compared to normocholesterolemic patients (38). 3-hydroxy-3-methylglutaryl coenzyme A (HMG-CoA) reductase is a rate-limiting enzyme of *de novo* cholesterol synthesis, and statins, as inhibitors of HMG-CoA reductase, prevent the conversion of HMG-CoA to L-mevalonate and inhibit downstream cholesterol biosynthesis (Figure 2). Cimato et al. treated human subjects with different statins, atorvastatin, pravastatin, and rosuvastatin, to vary cholesterol levels and analyzed the number of mobilized CD34^+^ HSPCs in the peripheral blood (39). They found a positive correlation between CD34^+^ HSPC number and both total and LDL-cholesterol levels. In addition, G-CSF and its upstream regulator IL-17 both correlated positively with LDL-cholesterol levels. Gao et al. studied the correlation between HDL and white blood cell levels in patients with coronary heart disease (27). They found negative correlations between HDL levels and both total white blood cell and neutrophil counts in the peripheral blood, and patients with low HDL-cholesterol had more mobilized Lineage^−^CD34^+^CD38^−^CD45RA^−/low^ HSCs in the peripheral blood as compared to the patients with normal HDL-cholesterol. Tolani et al. analyzed data from a clinical trial of rosuvastatin in children with heterozygous familial hypercholesterolemia and found that the children with the lowest HDL-cholesterol levels had higher monocyte counts in the peripheral blood, and there was an inverse correlation between HDL levels and monocyte percentage (40). Thus, increased cholesterol levels induce mobilization of not only mouse HSCs but human HSCs, which suggests that cholesterol level is a factor that should be considered when mobilizing HSCs for clinical transplantation.

## Roles of Cholesterol Metabolites in Hematopoiesis

### Sex Steroid Hormones

Estrogens and androgens are classically recognized as sex steroid hormones, and progestogen are recognized as a third class of sex steroid hormones. Each of these sex steroid hormones is synthesized from cholesterol, and the first and rate-limiting step of the steroidogenic pathway is the cleavage of the cholesterol side chain by P450scc (CYP11A1) to convert into pregnenolone (Figure 2) (41). Estrogens are produced in gonadal and extra-gonadal tissues. In females, 17β-estradiol (E2), a most potent estrogen, is produced primarily by theca and granulosa cells in the ovaries. Androstenedione is generated from cholesterol and is converted into testosterone by aromatase in theca cells, and they are further converted into E2 by aromatase in granulosa cells. Testosterone is the primary androgen secreted from Leydig cells in the testes, and small amounts are also secreted from theca cells in the ovaries. Progesterone is a critical progestogen to establish and maintain pregnancy. Progesterone is produced from cholesterol in the corpus luteum of the ovary during early pregnancy and the production is sustained by the placenta in humans and rodents. In addition to their well-recognized effects on reproductive tissues, the sex steroid hormones are also being recognized as having broad physiological effects on non-reproductive tissues, such as nervous, cardiovascular, skeletal, immune, and hematopoietic systems. It is known that females and males differ in innate and adaptive immune responses, and these sex-biased differences in the immune system contribute to variations in the prevalence of autoimmune diseases and malignancies, susceptibility to infectious diseases, and responses to vaccines (42).

### Androgens and Lympho-Hematopoiesis

Several studies have shown that androgens negatively regulate B lymphopoiesis. Castration of male mice leads to spleen enlargement and expansion of the B-cell population in the bone marrow and spleen (43–45). This effect is reversed by androgen replacement with either testosterone or dihydrotestosterone (DHT) (46). Androgen-resistant “testicular feminization” mutant male mice also show expansion of B-cell populations in the bone marrow and spleen (45, 47). In addition to the regulation of B lymphopoiesis, castrated mice and testicular feminization male mice also show thymic hypertrophy, which can be rescued by DHT administration (43, 45, 48–50). Experiments transplanting wild-type bone marrow cells into testicular feminization male mice suggest that androgen receptors expressed by bone marrow stromal cells or thymic epithelium modulate B-cell development or thymus size, respectively (50, 51). Velardi et al. showed that one mechanism by which androgens influence thymopoiesis is through direct inhibition of the Notch ligand *Dll4* in cortical thymic epithelial cells (52). Immune function progressively declines with age in mice and humans (53). In male mice, castration rejuvenates aged bone marrow and thymus, enhances peripheral T- and B-cell functions, and promotes immune recovery following chemotherapy-induced immunodepletion and HSC transplantation (54–62). These studies indicate that androgens are critical mediators of age-related lymphoid decline. Castration also enhances the recovery of bone marrow-resident HSCs after chemotherapy-induced immunodepletion (62). Khong et al. demonstrated that the number of HSCs marked by CD34^−^Flt3^−^LSK was significantly increased at 7 days after castration of 9-month-old mice as compared to sham-treated mice, and the repopulation potential during serial bone marrow transplantations was enhanced when using these mice as donors (63). Their gene expression analyses suggest that castration induces qualitative changes in both HSCs and their bone marrow environment.

### Estrogens and Lympho-Hematopoiesis

B lymphopoiesis in the bone marrow and T lymphopoiesis in thymus are drastically reduced during pregnancy (64, 65). Ovariectomy stimulates B lymphopoiesis and results in increased numbers of B cells in the bone marrow (66, 67), and it is reversed by administration of E2 (68). Genetically hypogonadal female mice which have a partial deletion of the hypothalamic gonadotropin-releasing hormone gene have a secondary deficiency in gonadal steroidogenesis and show expansion of B cell progenitors. Estrogen replacement with E2 reversed the increased numbers of B cell progenitors in these hypogonadal mice (69). Exogenous E2 treatment suppresses B cell development in both male and female mice (68, 70, 71). Very early lymphoid precursors marked by Lineage marker^−^IL-7Rα^+^c-Kit^lo^Terminal dexynucleotidyl transferase^+^ in the bone marrow are selectively depleted by exogenous E2 treatment (72). Both stromal-dependent and independent pathways of estrogen action on developing B cells have been postulated. It is proposed that bone marrow stromal cells expressing estrogen receptors mediate negative regulatory effects of E2 on early pre-B development (47, 73). A study of estrogen receptor α (ERα) male knockout mice by Thurmond et al. indicated that ERα is necessary for development of normal numbers of B cells in the bone marrow (74). They investigated the effect of E2 on lymphopoiesis by performing bone marrow transplantation using ERα-deficient mice as donors, recipients, or both, and treated with E2. They demonstrated that exogenous E2-induced alteration of B cell populations was primarily caused by a hematopoietic cell-intrinsic mechanism rather than by their environment.

Estrogens also regulate other hematopoietic lineages. Administration of a high-dose of estrogen induces anemia in rodents and dogs, regardless of their sex (75–77). Schroeder et al. reported that E2 sustained proliferation of erythroid progenitors from chick bone marrow, but E2 also caused erythroid differentiation arrest and blocked erythrocyte gene expression (78). Blobel et al. also showed that E2 added to the culture also reduced the number of erythroid progenitors from human bone marrow (79). They demonstrated that the transcriptional activity of GATA1, an erythroid master transcription factor that is necessary for full maturation of erythrocytes, was strongly repressed by direct binding of ER in an E2-dependent manner in NIH 3T3 and COS cell lines. The same group subsequently reported that inhibition of GATA1 activity by ER induced apoptosis in a murine Friend virus-induced erythroleukemia cell line (80). E2 treatment also stimulates the megakaryocyte colony formation potential of CD34^+^ human cord blood cells *in vitro* (81). E2 promotes megakaryocyte polyploidization and maturation via activation of ERβ accompanied by a significant upregulation of the expression of GATA1, which is also a key regulator of megakaryocyte differentiation (82). Differentiation of dendritic cells with characteristics of Langerhans cells from myeloid progenitors in culture, as induced by granulocyte-macrophage colony-stimulating factor (GM-CSF), is promoted by addition of E2 and inhibited by ER antagonists and ERα-deficiency (83). Interferon regulatory factor 4 (IRF4), a transcription factor induced by GM-CSF and critical for Langerhans cell development, is a target of ERα signaling during this process (84). In contrast, E2 significantly inhibits Flt3-ligand-induced plasmacytoid and conventional dendritic-cell differentiation in culture by decreasing numbers of viable differentiated cells (85). Thus, the effects of E2 are likely to be dependent on the cytokine pathways that might be operative in the steady state or during inflammation and disease.

### Estrogens Regulate HSC Division Rate

**Figure 3:**
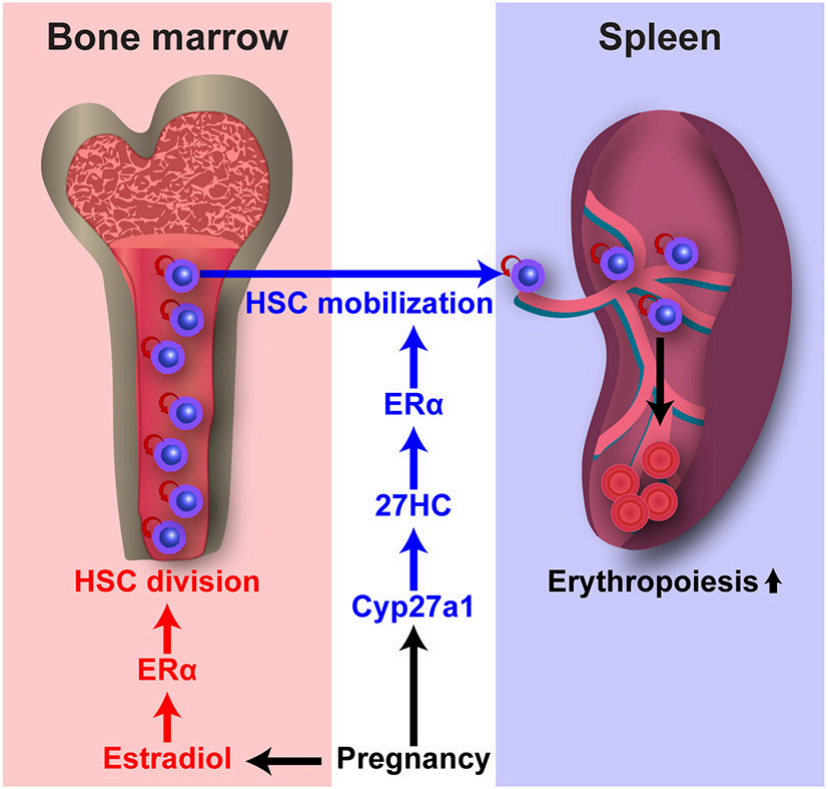
E2 and 27HC differentially regulate HSC division and mobilization during pregnancy. Pregnancy upregulates E2–ERα and 27HC–ERα signaling. E2–ERα signaling induces HSC division in the bone marrow. 27HC–ERα signaling induces HSC mobilization into the circulation and spleen, and augments splenic erythropoiesis.

Estrogens also regulate HSC behavior. In our previous study, we observed that HSCs divide more often in female mice as compared to male mice (14). Ovariectomy in females (which depletes both estrogens and progesterone) significantly reduced HSC division to male levels, while castration of males has no effect on HSC division. Conversely, administration of exogenous E2, but not progesterone or dihydrotestosterone, significantly increased HSC division in both female and male mice. Because E2 treatment also increased HSC division in ovariectomized female mice and castrated male mice, its action is independent of the gonads. Although HSC division rate was increased in untreated female mice as compared to male mice and in E2-treated mice of either sex as compared to vehicle-treated control mice, we did not observe an increase in absolute HSC numbers in those untreated female mice or E2-treated mice. Instead, we observed an increased generation of megakaryocyte-erythroid progenitors (MEPs) in female mice as compared to male mice, and increased splenic erythropoiesis in E2-treated mice of both sexes as compared to vehicle-treated control mice. Given that increased myeloid progenitors including MEPs may arise directly from the asymmetric division of HSCs (Figure 1) (86), our observations raise the possibility that the increased MEPs in female mice reflects increased asymmetric division of an HSC to produce one HSC and one MEP in response to E2. ERα, but not ERβ, is highly expressed by HSCs. Conditional deletion of *Esr1*, which encodes ERα, from hematopoietic cells significantly reduced the HSC division rate in female mice, but not in male mice. *Esr1*-deficient HSCs of both sexes were insensitive to exogenous E2 treatment. Moreover, E2 treatment of chimeric recipient mice transplanted with equal numbers of wild-type and *Esr1*-deficient hematopoietic cells revealed that E2 significantly induced division of wild-type HSCs but not *Esr1*-deficient HSCs in the same recipient mice, indicating that E2 acts directly on HSCs, rather than acting indirectly by stimulating secondary signals from other cells. E2 levels increase in females during pregnancy (87), when extramedullary hematopoiesis is induced to increase the production of red blood cells. Notably, pregnant mice exhibit significantly increased HSC division rate relative to non-pregnant female mice, and the deletion of *Esr1* in hematopoietic cells significantly reduced the normal increase in HSC division during pregnancy (Figure 3). Increased spleen size is observed during pregnancy in mice and humans (88, 89). In addition to the increased HSC division rate, we found that pregnant mice exhibited significantly increased cellularity, erythropoiesis, myelopoiesis, and HSC number in the spleen, indicating extramedullary hematopoiesis and HSC mobilization, and these processes also depend upon ERα function in HSCs. Pregnant mice also had increased absolute HSC numbers in the bone marrow, but deletion of *Esr1* in hematopoietic cells did not reverse this phenomenon, suggesting the existence of ERα-independent factor(s) that increase(s) HSC numbers in pregnant mice. Nevertheless, ERα signaling is critical for the induction of HSC division and mobilization to the spleen for the expansion of splenic erythropoiesis.

Illing et al. also reported the effects of E2 on HSC function (90). In this study, they treated mice with E2 at a dose of 0.24 mg/kg/day for 30 days, which is a higher dose and longer treatment than our study at a dose of 0.1 mg/kg/day for 7 days. In this condition, they observed a profound reduction in bone marrow cellularity by E2 treatment. E2 treatment caused more HSPCs to enter the S phase. They also observed an increased frequency of long-term reconstituting HSCs in E2-treated mice by performing bone marrow transplantation with a limiting dilution assay. However, donor-derived HSPCs of the bone marrow of the recipient mice after tertiary transplantation was decreased in the recipients that received bone marrow cells from E2-treated mice, suggesting exhaustion of reconstituting cells during serial transplantation. Deletion of *Esr1* reversed the reduction of bone marrow cellularity by E2 treatment, however it did not reverse the increased frequency of long-term reconstituting HSCs by E2 treatment. Sanchez-Aguilera et al. reported the effect of tamoxifen, a selective estrogen receptor modulator (SERM), on HSC function (91). Tamoxifen induced HSC division as well as apoptosis in MPPs, and these effects are mediated by ERα but not by ERβ. Tamoxifen treatment significantly reduced MPP number, but not HSC number, and compromised activation of hematopoiesis after chemotherapy. They observed increased expression of *Myc* in HSCs after tamoxifen treatment, and *Myc*-deficient MPPs did not undergo apoptosis upon tamoxifen treatment. Interestingly, female immunodeficient recipient mice support reconstitution of the blood system by transplanted human HSCs more efficiently than male immunodeficient recipient mice (92, 93). Since female mice have higher E2 levels than male mice and E2 induces HSC division, it is anticipated that higher E2 levels in female recipient mice will promote proliferation and differentiation of transplanted HSCs as compared to male recipient mice with very low E2 levels. The molecular mechanisms by which E2 regulates HSCs are not fully understood. Chapple et al. proposed that HSCs from mice treated with E2 had increased regenerative capacity after transplantation or irradiation (94). They demonstrated that E2–ERα signaling induced expression of *Ern1*, which encodes Ire1α, to activate the Ire1α-Xbp1 pathway of the unfolded protein response, and promoted resistance of HSCs against proteotoxic stress.

### 27-Hydroxycholesterol Regulates HSC Mobilization

Oxysterols are oxygenated derivatives of cholesterol and key substrates for bile acid synthesis (Figure 2) (16). In the classic pathway of bile acid synthesis, cholesterol is converted into 7α-hydroxycholesterol (7αHC) by cholesterol 7α-hydroxylase CYP7A1, a rate limiting step that occurs in the liver. In alternative pathways that occur primarily in extrahepatic tissues, cholesterol is converted into 24-hydroxycholesterol (24HC), 25-hydroxycholesterol (25HC), and 27-hydroxycholesterol (27HC) by cholesterol 24-hydroxylase CYP46A1, cholesterol 25-hydroxylase CH25H, and sterol 27-hydroxylase CYP27A1, respectively. Oxysterols are considered to be bioactive lipids and recent studies have started to reveal their important roles in both the hematopoietic and immune systems. 27HC is the most abundant circulating oxysterol and acts as an endogenous SERM which can bind to ERs and regulate their function (95). Plasma 27HC levels strongly correlate with total cholesterol levels (96), as 27HC is generated directly from cholesterol by the sterol 27-hydroxylase CYP27A1. Plasma 27HC levels are greatly reduced in *Cyp27a1*-deficient mice (97). CYP27A1 is abundant in the liver, but it is also expressed in extrahepatic tissues (16). Dietary or genetic changes that elevate 27HC levels modulate ER activity, thereby inhibiting vascular repair in cardiovascular disease (95), promoting ER-positive breast cancer growth (98, 99), and increasing the severity of atherosclerosis (100).

We have demonstrated that the increases in HSC division, HSC mobilization, and extramedullary hematopoiesis during pregnancy require ERα in HSCs, and E2 treatment induces HSC division as described above (14). Interestingly, our recent study revealed that treatment of mice with E2 did not increase HSC number in the spleen, indicating that E2 treatment does not induce HSC mobilization (101). In contrast, treatment with 27HC, another endogenous ER ligand, increased HSC number in the spleen but not HSC division in the bone marrow, indicating a role in inducing HSC mobilization. We demonstrated that the effect of 27HC on HSC mobilization is nullified by deletion of *Esr1* in hematopoietic cells, indicating that 27HC-induced HSC mobilization is dependent on ERα. Plasma cholesterol levels increase in humans during pregnancy (102). During pregnancy in mice, we observed significant increases in 27HC levels in HSPCs. *Cyp27a1*-deficient mice had significantly reduced HSC mobilization and extramedullary hematopoiesis during pregnancy, while the increased rate of HSC division in the bone marrow during pregnancy was not affected. These findings indicate that 27HC acts in concert with E2 to promote hematopoiesis during pregnancy by regulating ERα signaling in HSCs (Figure 3). As described above, increased cholesterol levels promote HSC mobilization in mice and humans, and increased HSC mobilization in *Abca1*^−/−^*Abcg1*^−/−^ mice is associated with elevated serum G-CSF levels (33). In our study, we observed that 27HC treatment significantly induced HSC mobilization in mice deficient for *Csf3*, which encodes G-CSF (101). Together, 27HC and G-CSF additively increased the numbers of colony-forming HSPCs in the peripheral blood. Therefore, 27HC and G-CSF likely act through distinct mechanisms to induce HSC mobilization. These findings suggest an alternative model that the ability of elevated cholesterol levels to promote HSC mobilization is mediated by increases in 27HC production, because treating mice with 27HC induces HSC mobilization and 27HC levels increase as cholesterol levels increase.

### E2, 27HC, and ERα

ERα is a nuclear receptor transcription factor and E2 is the most potent endogenous estrogen. Different ER ligands are known to have distinct effects on ERα-mediated regulation of gene expression, and ER ligands differ in their structures and their effects on ER conformation (103–106). For example, Wardell et al. used breast cancer cell lines to test E2 and five different synthetic ER ligands and observed different gene expression patterns regulated by different ER–ligand complexes (106). 27HC induces a unique conformational change in the ER that is different from that mediated by E2 and other ER ligands (107). Different ER–ligand complexes also engage functionally distinct coregulators by selective recruitment of coactivators and corepressors to activate and repress expression of target genes, respectively (108). Thus, as different ER ligands can act through the ERα to differentially regulate gene expression, this may explain why E2 and 27HC have distinct effects on HSCs even though both act through ERα (Figure 3). It will be interesting to explore the nature of the ERα target genes as well as the mechanism by which ERα function is differentially regulated by E2 and 27HC, two major endogenous ER ligands.

### Bile Acids

Bile acids are synthesized from cholesterol in the liver (Figure 2), secreted into the bile, and delivered to the lumen of the small intestine where they act as emulsifiers of dietary lipids, cholesterol, and fat-soluble vitamins (16). However, bile acids have additional roles (109). Tauroursodeoxycholic acid (TUDCA) serves as a chemical chaperone and reduces endoplasmic reticulum stress (110). HSCs are predisposed to apoptosis through misfolded protein accumulation in the endoplasmic reticulum caused by cellular stress that subsequently activates the unfolded protein response (111). Addition of TUDCA to cultured mouse HSCs alleviates endoplasmic reticulum stress and increases their capacity to reconstitute the hematopoietic system in recipient mice upon transplantation (112). In contrast to the quiescent state of adult HSCs in the bone marrow, HSCs undergo a rapid expansion in the fetal liver during development (113). Adult HSCs in the bone marrow have a lower rate of protein synthesis as compared to most other hematopoietic cells (114). Sigurdsson et al. reported that although fetal-liver HSCs had a higher rate of protein synthesis as compared to adult HSCs in the bone marrow, fetal-liver HSCs had lower expression of endoplasmic reticulum stress response genes (115). In addition to the role of CYP27A1 for side chain oxidation of cholesterol for the generation of 27HC to initiate the alternative acidic bile acid biosynthetic pathway, CYP27A1 also catalyzes another side chain oxidation after the ring modification step of both classic and alternative acidic bile acid synthesis pathways (Figure 2) (16). *Cyp27a1*-deficient mice have significantly decreased bile acids (97). Sigurdsson et al. also reported that fetuses in *Cyp27a1*-deficient mothers displayed significantly reduced levels of total bile acids as wells as secondary bile acids in the fetal liver, suggesting that maternal bile acids are transferred to the fetus during pregnancy (115). Interestingly, the livers of *Cyp27a1*-deficient fetuses in *Cyp27a1*-deficient mothers contained a significantly lower number of long-term reconstituting HSCs, as assessed by limiting dilution transplantation assay, and these fetal HSCs showed significantly higher levels of protein aggregation. These findings imply that bile acids enable fetal-liver HSCs to have a higher level of protein synthesis without activating a stress response, allowing expansion of the HSC pool during fetal development.

### 25-Hydroxycholesterol

25HC is generated directly from cholesterol by CH25H, and 25HC is further converted into 7α,25-dihydroxycholesterol (7α,25HC) by the oxysterol 7α-hydroxylase CYP7B1-mediated hydroxylation (Figure 2) (116). Expression of *Ch25h* is upregulated in macrophages and dendritic cells when they are exposed to various inflammatory mediators (117–120). 25HC augments the production of inflammatory cytokines in macrophages, and mediates feedback inhibition of *IL1b* expression and inflammasome activation in activated macrophages in the DNA sensor protein absent in melanoma 2 (AIM2)-dependent manner (121–124). 25HC is strongly induced following viral infection and by interferon, and it inhibits the replication of a wide range of enveloped viruses (125, 126). 25HC also promotes macrophage foam cell formation (127). Epstein-Barr virus-induced gene 2 (EBI2), a G protein-coupled receptor also known as GPR183, controls follicular B-cell migration and T-cell-dependent antibody production (128, 129). 7α,25HC acts as a ligand for EBI2 and directs migration of B cells in the spleen during the adaptive immune response (130, 131). EBI2 and 7α,25HC also regulate splenic CD4^+^ dendritic cells for positioning in marginal zone bridging channels to maintain their homeostasis and mount a response against certain antigens, and positioning of activated CD4 T cells at the interface of the follicle and T zone to interact with activated dendritic cells (132–134). Thus, 25HC broadly regulates innate and adaptive immune cell behavior. The 7α,25HC/EBI2 axis also regulates bone mass homeostasis (135). EBI2 is expressed in monocyte/osteoclast precursors, and 7α,25HC is secreted by osteoblasts. EBI2 guides osteoclast precursors toward bone surfaces by promoting their movement and positioning, which facilitates fusion of osteoclasts and enhances the development of large osteoclasts to maintain bone mass homeostasis.

## Cholesterol Synthesis/Metabolism and Hematologic Malignancies

Cholesterol metabolism is dysregulated in hematologic malignancies. The rate of cholesterol synthesis is higher in cells from acute myeloid leukemia (AML) patients as compared to healthy subjects (136). Hypocholesterolemia is frequently observed due to elevated LDL uptake by leukemia cells (137–139), but elevated cholesterol levels in leukemia cells have also been reported (140–143). Yvan-Charvet et al. reported that HFHC diet administered *Ldlr*^+/−^ mice transplanted with cellular cholesterol efflux pathway-deficient *Abca1*^−/−^*Abcg1*^−/−^ bone marrow cells displayed a myeloproliferative neoplasm (MPN)-like phenotype, and expression of an *APOA1* transgene that elevates HDL levels suppressed this phenotype (32). Thus, changes in intracellular cholesterol levels are associated with the development and maintenance of hematologic malignancies. Statins have cytotoxic effects in various types of malignant hematopoietic cells including AML (144–158), chronic myeloid leukemia (CML) (153, 159–161), MPNs (162), acute lymphocytic leukemia (ALL) (163, 164), chronic lymphocytic leukemia (CLL) (165–167), adult T-cell leukemia (ATL) (168), lymphoma (169, 170), and multiple myeloma (171–176). To identify compounds that can inhibit the stem cell activity of leukemia-initiating cells (LICs), Hartwell et al. performed a high-throughput screen in a bone marrow-mimicking culture system in which LICs expressing the *MLL-AF9* fusion oncogene were co-cultured with a bone marrow stromal cell line (177). Among the compounds that selectively inhibited LICs but not normal HSPCs, lovastatin also inhibited LIC stem cell activity in an *in vivo* bone marrow transplantation model. Although these reports demonstrate the effectiveness of statins, the mechanisms of their anticancer effects are not fully understood. Griner et al. reported that MPN-associated JAK2^V617F^ localized to lipid rafts, subdomains of the plasma membrane that contain protein receptors and a high concentration of cholesterol, and simvastatin inhibited this localization and JAK2^V617F^-dependent cell growth in MPN model cell lines (162). Simvastatin also inhibited erythroid colony formation of primary cells from MPN patients, but had no effect on cells from healthy individuals. Other than the cholesterol-lowering effect, inhibition of the mevalonate pathway by statins also reduces the levels of farnesyl pyrophosphate and geranylgeranyl pyrophosphate and thereby inhibits protein farnesylation and geranylgeranylation, modifications that are important for a variety of cellular processes including cell proliferation, survival, and migration (Figure 2). Thus, the anticancer effects of statins could also be rendered through changes in these other cellular processes (157, 158, 170, 176).

Among the metabolites of cholesterol, oxysterols such as 7βHC, 25HC, 7β,25HC, 7-ketocholestanol, and 7-ketocholestanol have cytotoxic effects on leukemia and lymphoma cells (178–183). Tsujioka et al. reported that DNA methyltransferase inhibitors induced *CH25H* expression with enhanced 25HC production and promoted apoptosis in leukemia and myelodysplastic syndrome (MDS) cell lines, while exogenous 25HC treatment suppressed cell growth of leukemia and MDS cell lines (184). Other than oxysterol, Sanchez-Aguilera et al. reported that tamoxifen treatment blocked development of *JAK*2^*V617F*^-induced MPN in mice and induced apoptosis of human MPN cells from patients with *JAK*2^*V617F*^ mutation in a xenograft model (91). They also demonstrated that tamoxifen treatment reduced leukemic burden in a mouse model of AML using mice transplanted with bone marrow cells expressing the *MLL-AF9* oncogene. Their findings have uncovered the potential role of estrogen signaling in leukemia and suggest the potential use of SERMs as a treatment for leukemia. The roles of cholesterol metabolites in hematologic malignancies are not yet fully explored, and further studies of cholesterol metabolites are expected to elucidate their roles in hematologic malignancies and their potential in preventing and treating hematologic malignancies.

## Conclusion and Future Directions

Cholesterol and its metabolites are now being recognized to have important roles in broad biological processes by regulating a wide variety of molecular machinery. Advances in understanding these molecular mechanisms will benefit human health. One potential clinical application of molecules that regulate or are regulated by cholesterol metabolism is to enhance mobilization of HSCs for transplantation. To enable efficient collection of mobilized HSCs from the peripheral blood for HSC transplantation, donors are treated with HSC-mobilizing agents such as G-CSF (185). However, a significant proportion of donors fail to reach the minimum HSC collection threshold required for transplantation using traditional strategies (186). The failure of mobilization can increase patient morbidity because patients cannot proceed to transplantation. Thus, advances in mobilization strategies that could increase the success of HSC collection without introducing additional side effects are needed to improve patient outcomes. For example, administration of 27HC enhances the mobilization of HSPCs by G-CSF (101). Identification of the genes downstream of 27HC-ERα signaling that mediate HSC mobilization may contribute to the development of new methods that improve the yield of mobilized HSCs for transplantation, while also offering an explanation for the long-standing observation that increased cholesterol levels are associated with increased HSC mobilization in mice and humans.

High blood cholesterol levels are associated with the development of atherosclerosis. Atherosclerosis is a progressive disease in which the inside of the artery become thick and stiff due to the buildup of the atheromatous plaque which consists of cholesterol, fat and other substances, and restricts blood flow and causes complications including myocardial infarction, peripheral artery disease, and stroke. In addition to lipids, various types of leukocytes also accumulate in the atheromatous plaque. Hypercholesterolemia causes monocytosis, and these monocytes give rise to macrophages which eventually turn into foam cells by ingesting LDL in the plaque, and promotes plaque growth and inflammation. Other than monocytes, diverse immune-cell subsets, such as neutrophils, mast cells, B and T lymphocytes, are associated with atherosclerosis [reviewed in (187, 188)]. Oxysterols are formed and accumulate in the plaque as a result of LDL oxidation due to the inflammatory response. Although atherosclerotic properties of oxysterols have been tested, it is still unclear whether oxysterols have pro- or anti-atherosclerotic properties [reviewed in (189)]. Estrogens also affect atherogenesis. Despite of reports that support the atheroprotective effects of estrogens, it is also controversial whether they have pro- or anti-atherosclerotic properties [reviewed in (190)]. After myocardial infarction, monocyte recruitment is increased, and sustained and accelerated atherosclerosis is observed in a mouse model. Interestingly, myocardial infarction causes HSPC mobilization into the spleen and sustains augmented monocytepoiesis, providing a possibility of novel therapy to mitigate progression of atherosclerosis (191, 192). Thus, cholesterol and its metabolites link hematopoiesis with cardiovascular health, and deciphering this link is critical for developing new targeted therapies.

The molecular mechanisms underlying the regulation of normal and malignant hematopoiesis by cholesterol and its metabolites are not yet fully understood. There are many drugs that target cholesterol synthetic and metabolic pathways, and further studies are expected to generate novel strategies for enhancing hematopoiesis, augmenting hematopoietic recovery after hematopoietic injuries, improving collection of mobilized HSCs for transplantation, and treating hematologic malignancies.

## Author Contributions

The author confirms being the sole contributor of this work and has approved it for publication.

### Conflict of Interest Statement

The author declares that the research was conducted in the absence of any commercial or financial relationships that could be construed as a potential conflict of interest.

## References

[1] Spangrude GJ Heimfeld S Weissman IL. Purification and characterization of mouse hematopoietic stem cells. Science. (1988) 241:58–62. 10.1126/science.2898810 2898810

[2] Okada S Nakauchi H Nagayoshi K Nishikawa S Miura Y Suda T. Enrichment and characterization of murine hematopoietic stem cells that express c-kit molecule. Blood. (1991) 78:1706–12. 1717068

[3] Ikuta K Weissman IL Evidence that hematopoietic stem cells express mouse c-kit but do not depend on steel factor for their generation. Proc Natl Acad Sci USA. (1992) 89:1502–6. 10.1073/pnas.89.4.1502 1371359 PMC48479

[4] Kiel MJ Yilmaz OH Iwashita T Terhorst C Morrison SJ. SLAM family receptors distinguish hematopoietic stem and progenitor cells and reveal endothelial niches for stem cells. Cell. (2005) 121:1109–21. 10.1016/j.cell.2005.05.026 15989959

[5] Oguro H Ding L Morrison SJ. SLAM family markers resolve functionally distinct subpopulations of hematopoietic stem cells and multipotent progenitors. Cell Stem Cell. (2013) 13:102–16. 10.1016/j.stem.2013.05.014 23827712 PMC3736853

[6] Osawa M Hanada K Hamada H Nakauchi H. Long-term lymphohematopoietic reconstitution by a single CD34-low/negative hematopoietic stem cell. Science. (1996) 273:242–5. 10.1126/science.273.5272.242 8662508

[7] Christensen JL Weissman IL. Flk-2 is a marker in hematopoietic stem cell differentiation: a simple method to isolate long-term stem cells. Proc Natl Acad Sci USA. (2001) 98:14541–6. 10.1073/pnas.261562798 11724967 PMC64718

[8] Sanjuan-Pla A Macaulay IC Jensen CT Woll PS Luis TC Mead A. Platelet-biased stem cells reside at the apex of the haematopoietic stem-cell hierarchy. Nature. (2013) 502:232–6. 10.1038/nature12495 23934107

[9] Notta F Doulatov S Laurenti E Poeppl A Jurisica I Dick JE. Isolation of single human hematopoietic stem cells capable of long-term multilineage engraftment. Science. (2011) 333:218–21. 10.1126/science.1201219 21737740

[10] Crane GM Jeffery E Morrison SJ. Adult haematopoietic stem cell niches. Nat Rev Immunol. (2017) 17:573–90. 10.1038/nri.2017.53 28604734

[11] Cheshier SH Prohaska SS Weissman IL. The effect of bleeding on hematopoietic stem cell cycling and self-renewal. Stem Cells Dev. (2007) 16:707–17. 10.1089/scd.2007.0017 17999593

[12] Randall TD Weissman IL. Phenotypic and functional changes induced at the clonal level in hematopoietic stem cells after 5-fluorouracil treatment. Blood. (1997) 89:3596–606. 9160664

[13] Baldridge MT King KY Boles NC Weksberg DC Goodell MA. Quiescent haematopoietic stem cells are activated by IFN-gamma in response to chronic infection. Nature. (2010) 465:793–7. 10.1038/nature09135 20535209 PMC2935898

[14] Nakada D Oguro H Levi BP Ryan N Kitano A Saitoh Y Takeichi M Wendt GR Morrison SJ. Oestrogen increases haematopoietic stem-cell self-renewal in females and during pregnancy. Nature. (2014) 505:555–8. 10.1038/nature12932 24451543 PMC4015622

[15] Ikonen E. Cellular cholesterol trafficking and compartmentalization. Nat Rev Mol Cell Biol. (2008) 9:125–38. 10.1038/nrm2336 18216769

[16] Russell DW. The enzymes, regulation, and genetics of bile acid synthesis. Annu Rev Biochem. (2003) 72:137–74. 10.1146/annurev.biochem.72.121801.161712 12543708

[17] Gomes AL Carvalho T Serpa J Torre C Dias S. Hypercholesterolemia promotes bone marrow cell mobilization by perturbing the SDF-1:CXCR4 axis. Blood. (2010) 115:3886–94. 10.1182/blood-2009-08-240580 20009035

[18] Karpova D Bonig H. Concise review: CXCR4/CXCL12 signaling in immature hematopoiesis–lessons from pharmacological and genetic models. Stem Cells. (2015) 33:2391–9. 10.1002/stem.2054 25966814

[19] Plump AS Smith JD Hayek T Aalto-Setala K Walsh A Verstuyft JG. Severe hypercholesterolemia and atherosclerosis in apolipoprotein E-deficient mice created by homologous recombination in ES cells. Cell. (1992) 71:343–53. 10.1016/0092-8674(92)90362-G 1423598

[20] Zhang SH Reddick RL Piedrahita JA Maeda N. Spontaneous hypercholesterolemia and arterial lesions in mice lacking apolipoprotein E. Science. (1992) 258:468–71. 10.1126/science.1411543 1411543

[21] Swirski FK Libby P Aikawa E Alcaide P Luscinskas FW Weissleder R Pittet MJ. Ly-6Chi monocytes dominate hypercholesterolemia-associated monocytosis and give rise to macrophages in atheromata. J Clin Invest. (2007) 117:195–205. 10.1172/JCI29950 17200719 PMC1716211

[22] Tacke F Alvarez D Kaplan TJ Jakubzick C Spanbroek R Llodra J. Monocyte subsets differentially employ CCR2, CCR5, and CX3CR1 to accumulate within atherosclerotic plaques. J Clin Invest. (2007) 117:185–94. 10.1172/JCI28549 17200718 PMC1716202

[23] Murphy AJ Akhtari M Tolani S Pagler T Bijl N Kuo CL. ApoE regulates hematopoietic stem cell proliferation, monocytosis, and monocyte accumulation in atherosclerotic lesions in mice. J Clin Invest. (2011) 121:4138–49. 10.1172/JCI57559 21968112 PMC3195472

[24] Feng Y Schouteden S Geenens R Van Duppen V Herijgers P Holvoet P. Hematopoietic stem/progenitor cell proliferation and differentiation is differentially regulated by high-density and low-density lipoproteins in mice. PLoS ONE. (2012) 7:e47286. 10.1371/journal.pone.0047286 23144813 PMC3492382

[25] Seijkens T Hoeksema MA Beckers L Smeets E Meiler S Levels J. Hypercholesterolemia-induced priming of hematopoietic stem and progenitor cells aggravates atherosclerosis. FASEB J. (2014) 28:2202–13. 10.1096/fj.13-243105 24481967

[26] Rigotti A Trigatti BL Penman M Rayburn H Herz J Krieger M. A targeted mutation in the murine gene encoding the high density lipoprotein (HDL) receptor scavenger receptor class B type I reveals its key role in HDL metabolism. Proc Natl Acad Sci USA. (1997) 94:12610–5. 10.1073/pnas.94.23.12610 9356497 PMC25055

[27] Gao M Zhao D Schouteden S Sorci-Thomas MG Van Veldhoven PP Eggermont K. Regulation of high-density lipoprotein on hematopoietic stem/progenitor cells in atherosclerosis requires scavenger receptor type BI expression. Arterioscler Thromb Vasc Biol. (2014) 34:1900–9. 10.1161/ATVBAHA.114.304006 24969774 PMC4140992

[28] Ito K Hirao A Arai F Takubo K Matsuoka S Miyamoto K. Reactive oxygen species act through p38 MAPK to limit the lifespan of hematopoietic stem cells. Nat Med. (2006) 12:446–51. 10.1038/nm1388 16565722

[29] Tie G Messina KE Yan J Messina JA Messina LM. Hypercholesterolemia induces oxidant stress that accelerates the ageing of hematopoietic stem cells. J Am Heart Assoc. (2014) 3:e000241. 10.1161/JAHA.113.000241 24470519 PMC3959695

[30] Westerterp M Bochem AE Yvan-Charvet L Murphy AJ Wang N Tall AR. ATP-binding cassette transporters, atherosclerosis, and inflammation. Circ Res. (2014) 114:157–70. 10.1161/CIRCRESAHA.114.300738 24385509

[31] Yvan-Charvet L Ranalletta M Wang N Han S Terasaka N Li R Welch C Tall AR. Combined deficiency of ABCA1 and ABCG1 promotes foam cell accumulation and accelerates atherosclerosis in mice. J Clin Invest. (2007) 117:3900–8. 10.1172/JCI33372 17992262 PMC2066200

[32] Yvan-Charvet L Pagler T Gautier EL Avagyan S Siry RL Han S. ATP-binding cassette transporters and HDL suppress hematopoietic stem cell proliferation. Science. (2010) 328:1689–93. 10.1126/science.1189731 20488992 PMC3032591

[33] Westerterp M Gourion-Arsiquaud S Murphy AJ Shih A Cremers S Levine RL. Regulation of hematopoietic stem and progenitor cell mobilization by cholesterol efflux pathways. Cell Stem Cell. (2012) 11:195–206. 10.1016/j.stem.2012.04.024 22862945 PMC3413200

[34] Fossiez F Djossou O Chomarat P Flores-Romo L Ait-Yahia S Maat C. T cell interleukin-17 induces stromal cells to produce proinflammatory and hematopoietic cytokines. J Exp Med. (1996) 183:2593–603. 10.1084/jem.183.6.2593 8676080 PMC2192621

[35] Stark MA Huo Y Burcin TL Morris MA Olson TS Ley K. Phagocytosis of apoptotic neutrophils regulates granulopoiesis via IL-23 and IL-17. Immunity. (2005) 22:285–94. 10.1016/j.immuni.2005.01.011 15780986

[36] Semerad CL Christopher MJ Liu F Short B Simmons PJ Winkler I. G-CSF potently inhibits osteoblast activity and CXCL12 mRNA expression in the bone marrow. Blood. (2005) 106:3020–7. 10.1182/blood-2004-01-0272 16037394 PMC1895331

[37] Westerterp M Murphy AJ Wang M Pagler TA Vengrenyuk Y Kappus MS. Deficiency of ATP-binding cassette transporters A1 and G1 in macrophages increases inflammation and accelerates atherosclerosis in mice. Circ Res. (2013) 112:1456–65. 10.1161/CIRCRESAHA.113.301086 23572498 PMC3839866

[38] Crysandt M Hilgers RD von Hobe S Eisert A Jost E Panse J. Hypercholesterolemia and its association with enhanced stem cell mobilization and harvest after high-dose cyclophosphamide+G-CSF. Bone Marrow Transplant. (2011) 46:1426–9. 10.1038/bmt.2010.327 21217788

[39] Cimato TR Palka BA Lang JK Young RF. LDL cholesterol modulates human CD34+ HSPCs through effects on proliferation and the IL-17 G-CSF axis. PLoS ONE. (2013) 8:e73861. 10.1371/journal.pone.0073861 23991206 PMC3753239

[40] Tolani S Pagler TA Murphy AJ Bochem AE Abramowicz S Welch C. Hypercholesterolemia and reduced HDL-C promote hematopoietic stem cell proliferation and monocytosis: studies in mice and FH children. Atherosclerosis. (2013) 229:79–85. 10.1016/j.atherosclerosis.2013.03.031 23684512 PMC3691284

[41] Miller WL Auchus RJ. The molecular biology, biochemistry, and physiology of human steroidogenesis and its disorders. Endocr Rev. (2011) 32:81–151. 10.1210/er.2010-0013 21051590 PMC3365799

[42] Klein SL Flanagan KL. Sex differences in immune responses. Nat Rev Immunol. (2016) 16:626–38. 10.1038/nri.2016.90 27546235

[43] Viselli SM Stanziale S Shults K Kovacs WJ Olsen NJ. Castration alters peripheral immune function in normal male mice. Immunology. (1995) 84:337–42. 7751013 PMC1415104

[44] Wilson CA Mrose SA Thomas DW. Enhanced production of B lymphocytes after castration. Blood. (1995) 85:1535–9. 7534134

[45] Ellis TM Moser MT Le PT Flanigan RC Kwon ED. Alterations in peripheral B cells and B cell progenitors following androgen ablation in mice. Int Immunol. (2001) 13:553–8. 10.1093/intimm/13.4.553 11282994

[46] Viselli SM Reese KR Fan J Kovacs WJ Olsen NJ. Androgens alter B cell development in normal male mice. Cell Immunol. (1997) 182:99–104. 10.1006/cimm.1997.1227 9514700

[47] Smithson G Couse JF Lubahn DB Korach KS Kincade PW. The role of estrogen receptors and androgen receptors in sex steroid regulation of B lymphopoiesis. J Immunol. (1998) 161:27–34. 9647203

[48] Olsen NJ Watson MB Kovacs WJ. Studies of immunological function in mice with defective androgen action. Distinction between alterations in immune function due to hormonal insensitivity and alterations due to other genetic factors. Immunology. (1991) 73:52–7. 2045127 PMC1384517

[49] Olsen NJ Watson MB Henderson GS Kovacs WJ. Androgen deprivation induces phenotypic and functional changes in the thymus of adult male mice. Endocrinology. (1991) 129:2471–6. 10.1210/endo-129-5-2471 1834454

[50] Olsen NJ Olson G Viselli SM Gu X Kovacs WJ. Androgen receptors in thymic epithelium modulate thymus size and thymocyte development. Endocrinology. (2001) 142:1278–83. 10.1210/endo.142.3.8032 11181545

[51] Olsen NJ Gu X Kovacs WJ. Bone marrow stromal cells mediate androgenic suppression of B lymphocyte development. J Clin Invest. (2001) 108:1697–704. 10.1172/JCI200113183 11733565 PMC200984

[52] Velardi E Tsai JJ Holland AM Wertheimer T Yu VW Zakrzewski JL. Sex steroid blockade enhances thymopoiesis by modulating Notch signaling. J Exp Med. (2014) 211:2341–9. 10.1084/jem.20131289 25332287 PMC4235646

[53] Dorshkind K Montecino-Rodriguez E Signer RA. The ageing immune system: is it ever too old to become young again? Nat Rev Immunol. (2009) 9:57–62. 10.1038/nri2471 19104499

[54] Dudakov JA Goldberg GL Reiseger JJ Chidgey AP Boyd RL. Withdrawal of sex steroids reverses age- and chemotherapy-related defects in bone marrow lymphopoiesis. J Immunol. (2009) 182:6247–60. 10.4049/jimmunol.0802446 19414778

[55] Fitzpatrick FT Kendall MD Wheeler MJ Adcock IM Greenstein BD. Reappearance of thymus of ageing rats after orchidectomy. J Endocrinol. (1985) 106:R17–9. 10.1677/joe.0.106R017 4045335

[56] Goldberg GL Sutherland JS Hammet MV Milton MK Heng TS Chidgey AP Boyd RL. Sex steroid ablation enhances lymphoid recovery following autologous hematopoietic stem cell transplantation. Transplantation. (2005) 80:1604–13. 10.1097/01.tp.0000183962.64777.da 16371932

[57] Goldberg GL Alpdogan O Muriglan SJ Hammett MV Milton MK Eng JM. Enhanced immune reconstitution by sex steroid ablation following allogeneic hemopoietic stem cell transplantation. J Immunol. (2007) 178:7473–84. 10.4049/jimmunol.178.11.7473 17513799

[58] Goldberg GL King CG Nejat RA Suh DY Smith OM Bretz JC. Luteinizing hormone-releasing hormone enhances T cell recovery following allogeneic bone marrow transplantation. J Immunol. (2009) 182:5846–54. 10.4049/jimmunol.0801458 19380833 PMC2760441

[59] Greenstein BD Fitzpatrick FT Adcock IM Kendall MD Wheeler MJ. Reappearance of the thymus in old rats after orchidectomy: inhibition of regeneration by testosterone. J Endocrinol. (1986) 110:417–22. 10.1677/joe.0.1100417 3760740

[60] Sutherland JS Goldberg GL Hammett MV Uldrich AP Berzins SP Heng TS. Activation of thymic regeneration in mice and humans following androgen blockade. J Immunol. (2005) 175:2741–53. 10.4049/jimmunol.175.4.2741 16081852

[61] Goldberg GL Dudakov JA Reiseger JJ Seach N Ueno T Vlahos K. Sex steroid ablation enhances immune reconstitution following cytotoxic antineoplastic therapy in young mice. J Immunol. (2010) 184:6014–24. 10.4049/jimmunol.0802445 20483779

[62] Dudakov JA Goldberg GL Reiseger JJ Vlahos K Chidgey AP Boyd RL. Sex steroid ablation enhances hematopoietic recovery following cytotoxic antineoplastic therapy in aged mice. J Immunol. (2009) 183:7084–94. 10.4049/jimmunol.0900196 19890044

[63] Khong DM Dudakov JA Hammett MV Jurblum MI Khong SM Goldberg GL. Enhanced hematopoietic stem cell function mediates immune regeneration following sex steroid blockade. Stem Cell Reports. (2015) 4:445–58. 10.1016/j.stemcr.2015.01.018 25733018 PMC4375937

[64] Rijhsinghani AG Bhatia SK Tygrett LT Waldschmidt TJ. Effect of pregnancy on thymic T cell development. Am J Reprod Immunol. (1996) 35:523–8. 10.1111/j.1600-0897.1996.tb00052.x 8792935

[65] Medina KL Smithson G Kincade PW. Suppression of B lymphopoiesis during normal pregnancy. J Exp Med. (1993) 178:1507–15. 10.1084/jem.178.5.1507 8228804 PMC2191236

[66] Erben RG Raith S Eberle J Stangassinger M. Ovariectomy augments B lymphopoiesis and generation of monocyte-macrophage precursors in rat bone marrow. Am J Physiol. (1998) 274(3 Pt 1):E476–83. 10.1152/ajpendo.1998.274.3.E476 9530131

[67] Miyaura C Onoe Y Inada M Maki K Ikuta K Ito M Suda T. Increased B-lymphopoiesis by interleukin 7 induces bone loss in mice with intact ovarian function: similarity to estrogen deficiency. Proc Natl Acad Sci USA. (1997) 94:9360–5. 10.1073/pnas.94.17.9360 9256487 PMC23193

[68] Masuzawa T Miyaura C Onoe Y Kusano K Ohta H Nozawa S. Estrogen deficiency stimulates B lymphopoiesis in mouse bone marrow. J Clin Invest. (1994) 94:1090–7. 10.1172/JCI117424 8083350 PMC295170

[69] Smithson G Beamer WG Shultz KL Christianson SW Shultz LD Kincade PW. Increased B lymphopoiesis in genetically sex steroid-deficient hypogonadal (hpg) mice. J Exp Med. (1994) 180:717–20. 10.1084/jem.180.2.717 8046347 PMC2191601

[70] Medina KL Kincade PW. Pregnancy-related steroids are potential negative regulators of B lymphopoiesis. Proc Natl Acad Sci USA. (1994) 91:5382–6. 10.1073/pnas.91.12.5382 8202495 PMC43999

[71] Medina KL Strasser A Kincade PW. Estrogen influences the differentiation, proliferation, and survival of early B-lineage precursors. Blood. (2000) 95:2059–67. 10706875

[72] Medina KL Garrett KP Thompson LF Rossi MI Payne KJ Kincade PW. Identification of very early lymphoid precursors in bone marrow and their regulation by estrogen. Nat Immunol. (2001) 2:718–24. 10.1038/90659 11477408

[73] Smithson G Medina K Ponting I Kincade PW. Estrogen suppresses stromal cell-dependent lymphopoiesis in culture. J Immunol. (1995) 155:3409–17. 7561035

[74] Thurmond TS Murante FG Staples JE Silverstone AE Korach KS Gasiewicz TA. Role of estrogen receptor alpha in hematopoietic stem cell development and B lymphocyte maturation in the male mouse. Endocrinology. (2000) 141:2309–18. 10.1210/endo.141.7.7560 10875230

[75] Dukes PP Goldwasser E. Inhibition of erythropoiesis by estrogens. Endocrinology. (1961) 69:21–9. 10.1210/endo-69-1-21 13724883

[76] Mirand EA Gordon AS. Mechanism of estrogen action in erythropoiesis. Endocrinology. (1966) 78:325–32. 10.1210/endo-78-2-325 5904769

[77] Crafts RG. The effects of estrogens on the bone marrow of adult female dogs. Blood. (1948) 3:276–85. 18902575

[78] Schroeder C Gibson L Nordstrom C Beug H. The estrogen receptor cooperates with the TGF alpha receptor (c-erbB) in regulation of chicken erythroid progenitor self-renewal. EMBO J. (1993) 12:951–60. 10.1002/j.1460-2075.1993.tb05736.x 8458346 PMC413296

[79] Blobel GA Sieff CA Orkin SH. Ligand-dependent repression of the erythroid transcription factor GATA-1 by the estrogen receptor. Mol Cell Biol. (1995) 15:3147–53. 10.1128/MCB.15.6.3147 7760810 PMC230546

[80] Blobel GA Orkin SH. Estrogen-induced apoptosis by inhibition of the erythroid transcription factor GATA-1. Mol Cell Biol. (1996) 16:1687–94. 10.1128/MCB.16.4.1687 8657144 PMC231155

[81] Bord S Frith E Ireland DC Scott MA Craig JI Compston JE. Estrogen stimulates differentiation of megakaryocytes and modulates their expression of estrogen receptors alpha and beta. J Cell Biochem. (2004) 92:249–57. 10.1002/jcb.20035 15108352

[82] Du C Xu Y Yang K Chen S Wang X Wang S. Estrogen promotes megakaryocyte polyploidization via estrogen receptor beta-mediated transcription of GATA1. Leukemia. (2017) 31:945–56. 10.1038/leu.2016.285 27748371

[83] Paharkova-Vatchkova V Maldonado R Kovats S. Estrogen preferentially promotes the differentiation of CD11c+ CD11b(intermediate) dendritic cells from bone marrow precursors. J Immunol. (2004) 172:1426–36. 10.4049/jimmunol.172.3.1426 14734718

[84] Carreras E Turner S Frank MB Knowlton N Osban J Centola M. Estrogen receptor signaling promotes dendritic cell differentiation by increasing expression of the transcription factor IRF4. Blood. (2010) 115:238–46. 10.1182/blood-2009-08-236935 19880499 PMC2808152

[85] Carreras E Turner S Paharkova-Vatchkova V Mao A Dascher C Kovats S. Estradiol acts directly on bone marrow myeloid progenitors to differentially regulate GM-CSF or Flt3 ligand-mediated dendritic cell differentiation. J Immunol. (2008) 180:727–38. 10.4049/jimmunol.180.2.727 18178810

[86] Yamamoto R Morita Y Ooehara J Hamanaka S Onodera M Rudolph KL. Clonal analysis unveils self-renewing lineage-restricted progenitors generated directly from hematopoietic stem cells. Cell. (2013) 154:1112–26. 10.1016/j.cell.2013.08.007 23993099

[87] Mahendroo MS Cala KM Landrum DP Russell DW. Fetal death in mice lacking 5alpha-reductase type 1 caused by estrogen excess. Mol Endocrinol. (1997) 11:917–27. 9178751 10.1210/mend.11.7.9933

[88] Sheehan HL Falkiner NM. Splenic aneurysm and splenic enlargement in pregnancy. Br Med J. (1948) 2:1105. 10.1136/bmj.2.4590.1105 18103296 PMC2092335

[89] Fowler JH Nash DJ. Erythropoiesis in the spleen and bone marrow of the pregnant mouse. Dev Biol. (1968) 18:331–53. 10.1016/0012-1606(68)90045-6 5752783

[90] Illing A Liu P Ostermay S Schilling A de Haan G Krust A. Estradiol increases hematopoietic stem and progenitor cells independent of its actions on bone. Haematologica. (2012) 97:1131–5. 10.3324/haematol.2011.052456 22371175 PMC3409808

[91] Sanchez-Aguilera A Arranz L Martin-Perez D Garcia-Garcia A Stavropoulou V. Estrogen signaling selectively induces apoptosis of hematopoietic progenitors and myeloid neoplasms without harming steady-state hematopoiesis. Cell Stem Cell. (2014) 15:791–804. 10.1016/j.stem.2014.11.002 25479752

[92] Notta F Doulatov S Dick JE. Engraftment of human hematopoietic stem cells is more efficient in female NOD/SCID/IL-2Rgc-null recipients. Blood. (2010) 115:3704–7. 10.1182/blood-2009-10-249326 20207983

[93] Miller PH Rabu G MacAldaz M Knapp DJ Cheung AM Dhillon K. Analysis of parameters that affect human hematopoietic cell outputs in mutant c-kit-immunodeficient mice. Exp Hematol. (2017) 48:41–9. 10.1016/j.exphem.2016.12.012 28087429 PMC5926796

[94] Chapple RH Hu T Tseng YJ Liu L Kitano A Luu V. ERalpha promotes murine hematopoietic regeneration through the Ire1alpha-mediated unfolded protein response. Elife. (2018) 7. 10.7554/eLife.31159 29451493 PMC5829925

[95] Umetani M Domoto H Gormley AK Yuhanna IS Cummins CL Javitt NB Mangelsdorf DJ. 27-Hydroxycholesterol is an endogenous SERM that inhibits the cardiovascular effects of estrogen. Nat Med. (2007) 13:1185–92. 10.1038/nm1641 17873880

[96] Karuna R Holleboom AG Motazacker MM Kuivenhoven JA Frikke-Schmidt R Tybjaerg-Hansen A. Plasma levels of 27-hydroxycholesterol in humans and mice with monogenic disturbances of high density lipoprotein metabolism. Atherosclerosis. (2011) 214:448–55. 10.1016/j.atherosclerosis.2010.10.042 21130455

[97] Rosen H Reshef A Maeda N Lippoldt A Shpizen S Triger L. Markedly reduced bile acid synthesis but maintained levels of cholesterol and vitamin D metabolites in mice with disrupted sterol 27-hydroxylase gene. J Biol Chem. (1998) 273:14805–12. 10.1074/jbc.273.24.14805 9614081

[98] Wu Q Ishikawa T Sirianni R Tang H McDonald JG Yuhanna IS Shaul PW. 27-Hydroxycholesterol promotes cell-autonomous, ER-positive breast cancer growth. Cell Rep. (2013) 5:637–45. 10.1016/j.celrep.2013.10.006 24210818 PMC3950897

[99] Nelson ER Wardell SE Jasper JS Park S Suchindran S Howe MK. 27-Hydroxycholesterol links hypercholesterolemia and breast cancer pathophysiology. Science. (2013) 342:1094–8. 10.1126/science.1241908 24288332 PMC3899689

[100] Umetani M Ghosh P Ishikawa T Umetani J Ahmed M Mineo C. The cholesterol metabolite 27-hydroxycholesterol promotes atherosclerosis via proinflammatory processes mediated by estrogen receptor alpha. Cell Metab. (2014) 20:172–82. 10.1016/j.cmet.2014.05.013 24954418 PMC4098728

[101] Oguro H McDonald JG Zhao Z Umetani M Shaul PW Morrison SJ. 27-Hydroxycholesterol induces hematopoietic stem cell mobilization and extramedullary hematopoiesis during pregnancy. J Clin Invest. (2017) 127:3392–401. 10.1172/JCI94027 28783041 PMC5669562

[102] Ordovas JM Pocovi M Grande F. Plasma lipids and cholesterol esterification rate during pregnancy. Obstet Gynecol. (1984) 63:20–5. 6691013

[103] Schwartz JA Skafar DF. Ligand-mediated modulation of estrogen receptor conformation by estradiol analogs. Biochemistry. (1993) 32:10109–15. 10.1021/bi00089a029 8399136

[104] Paige LA Christensen DJ Gron H Norris JD Gottlin EB Padilla KM. Estrogen receptor (ER) modulators each induce distinct conformational changes in ER alpha and ER beta. Proc Natl Acad Sci USA. (1999) 96:3999–4004. 10.1073/pnas.96.7.3999 10097152 PMC22409

[105] Bruning JB Parent AA Gil G Zhao M Nowak J Pace MC. Coupling of receptor conformation and ligand orientation determine graded activity. Nat Chem Biol. (2010) 6:837–43. 10.1038/nchembio.451 20924370 PMC2974172

[106] Wardell SE Kazmin D McDonnell DP. Research resource: transcriptional profiling in a cellular model of breast cancer reveals functional and mechanistic differences between clinically relevant SERM and between SERM/estrogen complexes. Mol Endocrinol. (2012) 26:1235–48. 10.1210/me.2012-1031 22570330 PMC3385791

[107] DuSell CD Umetani M Shaul PW Mangelsdorf DJ McDonnell DP. 27-hydroxycholesterol is an endogenous selective estrogen receptor modulator. Mol Endocrinol. (2008) 22:65–77. 10.1210/me.2007-0383 17872378 PMC2194632

[108] Feng Q O'Malley BW. Nuclear receptor modulation–role of coregulators in selective estrogen receptor modulator (SERM) actions. Steroids. (2014) 90:39–43. 10.1016/j.steroids.2014.06.008 24945111 PMC4192004

[109] de Aguiar Vallim TQ Tarling EJ Edwards PA. Pleiotropic roles of bile acids in metabolism. Cell Metab. (2013) 17:657–69. 10.1016/j.cmet.2013.03.013 23602448 PMC3654004

[110] Ozcan U Yilmaz E Ozcan L Furuhashi M Vaillancourt E Smith RO Gorgun CZ Hotamisligil GS. chemical chaperones reduce ER stress and restore glucose homeostasis in a mouse model of type 2 diabetes. Science. (2006) 313:1137–40. 10.1126/science.1128294 16931765 PMC4741373

[111] van Galen P Kreso A Wienholds E Laurenti E Eppert K Lechman ER. Reduced lymphoid lineage priming promotes human hematopoietic stem cell expansion. Cell Stem Cell. (2014) 14:94–106. 10.1016/j.stem.2013.11.021 24388174

[112] Miharada K Sigurdsson V Karlsson S. Dppa5 improves hematopoietic stem cell activity by reducing endoplasmic reticulum stress. Cell Rep. (2014) 7:1381–92. 10.1016/j.celrep.2014.04.056 24882002

[113] Ema H Nakauchi H. Expansion of hematopoietic stem cells in the developing liver of a mouse embryo. Blood. (2000) 95:2284–8. 10733497

[114] Signer RA Magee JA Salic A Morrison SJ. Haematopoietic stem cells require a highly regulated protein synthesis rate. Nature. (2014) 509:49–54. 10.1038/nature13035 24670665 PMC4015626

[115] Sigurdsson V Takei H Soboleva S Radulovic V Galeev R Siva K. Bile acids protect expanding hematopoietic stem cells from unfolded protein stress in fetal liver. Cell Stem Cell. (2016) 18:522–32. 10.1016/j.stem.2016.01.002 26831518

[116] Cyster JG Dang EV Reboldi A Yi T. 25-Hydroxycholesterols in innate and adaptive immunity. Nat Rev Immunol. (2014) 14:731–43. 10.1038/nri3755 25324126

[117] Bauman DR Bitmansour AD McDonald JG Thompson BM Liang G Russell DW. 25-Hydroxycholesterol secreted by macrophages in response to Toll-like receptor activation suppresses immunoglobulin A production. Proc Natl Acad Sci USA. (2009) 106:16764–9. 10.1073/pnas.0909142106 19805370 PMC2757821

[118] Park K Scott AL. Cholesterol 25-hydroxylase production by dendritic cells and macrophages is regulated by type I interferons. J Leukoc Biol. (2010) 88:1081–7. 10.1189/jlb.0610318 20699362 PMC2996899

[119] Diczfalusy U Olofsson KE Carlsson AM Gong M Golenbock DT Rooyackers O. Marked upregulation of cholesterol 25-hydroxylase expression by lipopolysaccharide. J Lipid Res. (2009) 50:2258–64. 10.1194/jlr.M900107-JLR200 19502589 PMC2759831

[120] Zou T Garifulin O Berland R Boyartchuk VL. Listeria monocytogenes infection induces prosurvival metabolic signaling in macrophages. Infect Immun. (2011) 79:1526–35. 10.1128/IAI.01195-10 21263022 PMC3067555

[121] Reboldi A Dang EV McDonald JG Liang G Russell DW Cyster JG. Inflammation. 25-Hydroxycholesterol suppresses interleukin-1-driven inflammation downstream of type I interferon. Science. (2014) 345:679–84. 10.1126/science.1254790 25104388 PMC4289637

[122] Gold ES Diercks AH Podolsky I Podyminogin RL Askovich PS Treuting PM Aderem A. 25-Hydroxycholesterol acts as an amplifier of inflammatory signaling. Proc Natl Acad Sci USA. (2014) 111:10666–71. 10.1073/pnas.1404271111 24994901 PMC4115544

[123] Koarai A Yanagisawa S Sugiura H Ichikawa T Kikuchi T Furukawa K. 25-Hydroxycholesterol enhances cytokine release and Toll-like receptor 3 response in airway epithelial cells. Respir Res. (2012) 13:63. 10.1186/1465-9921-13-63 22849850 PMC3460764

[124] Dang EV McDonald JG Russell DW Cyster JG. Oxysterol restraint of cholesterol synthesis prevents AIM2 inflammasome activation. Cell. (2017) 171:1057–71 e11. 10.1016/j.cell.2017.09.029 29033131 PMC5693620

[125] Blanc M Hsieh WY Robertson KA Kropp KA Forster T Shui G. The transcription factor STAT-1 couples macrophage synthesis of 25-hydroxycholesterol to the interferon antiviral response. Immunity. (2013) 38:106–18. 10.1016/j.immuni.2012.11.004 23273843 PMC3556782

[126] Liu SY Aliyari R Chikere K Li G Marsden MD Smith JK. Interferon-inducible cholesterol-25-hydroxylase broadly inhibits viral entry by production of 25-hydroxycholesterol. Immunity. (2013) 38:92–105. 10.1016/j.immuni.2012.11.005 23273844 PMC3698975

[127] Gold ES Ramsey SA Sartain MJ Selinummi J Podolsky I Rodriguez DJ. ATF3 protects against atherosclerosis by suppressing 25-hydroxycholesterol-induced lipid body formation. J Exp Med. (2012) 209:807–17. 10.1084/jem.20111202 22473958 PMC3328364

[128] Gatto D Paus D Basten A Mackay CR Brink R. Guidance of B cells by the orphan G protein-coupled receptor EBI2 shapes humoral immune responses. Immunity. (2009) 31:259–69. 10.1016/j.immuni.2009.06.016 19615922

[129] Pereira JP Kelly LM Xu Y Cyster JG. EBI2 mediates B cell segregation between the outer and centre follicle. Nature. (2009) 460:1122–6. 10.1038/nature08226 19597478 PMC2809436

[130] Liu C Yang XV Wu J Kuei C Mani NS Zhang L. Oxysterols direct B-cell migration through EBI2. Nature. (2011) 475:519–23. 10.1038/nature10226 21796211

[131] Hannedouche S Zhang J Yi T Shen W Nguyen D Pereira JP. Oxysterols direct immune cell migration via EBI2. Nature. (2011) 475:524–7. 10.1038/nature10280 21796212 PMC4297623

[132] Gatto D Wood K Caminschi I Murphy-Durland D Schofield P Christ D. The chemotactic receptor EBI2 regulates the homeostasis, localization and immunological function of splenic dendritic cells. Nat Immunol. (2013) 14:446–53. 10.1038/ni.2555 23502855

[133] Yi T Cyster JG. EBI2-mediated bridging channel positioning supports splenic dendritic cell homeostasis and particulate antigen capture. Elife. (2013) 2:e00757. 10.7554/eLife.00757 23682316 PMC3654440

[134] Li J Lu E Yi T Cyster JG. EBI2 augments Tfh cell fate by promoting interaction with IL-2-quenching dendritic cells. Nature. (2016) 533:110–4. 10.1038/nature17947 27147029 PMC4883664

[135] Nevius E Pinho F Dhodapkar M Jin H Nadrah K Horowitz MC. Oxysterols and EBI2 promote osteoclast precursor migration to bone surfaces and regulate bone mass homeostasis. J Exp Med. (2015) 212:1931–46. 10.1084/jem.20150088 26438360 PMC4612084

[136] Ho YK Smith RG Brown MS Goldstein JL. Low-density lipoprotein (LDL) receptor activity in human acute myelogenous leukemia cells. Blood. (1978) 52:1099–114. 214187

[137] Vitols S Angelin B Ericsson S Gahrton G Juliusson G Masquelier M. Uptake of low density lipoproteins by human leukemic cells *in vivo*: relation to plasma lipoprotein levels and possible relevance for selective chemotherapy. Proc Natl Acad Sci USA. (1990) 87:2598–602. 10.1073/pnas.87.7.2598 2320578 PMC53737

[138] Vitols S Gahrton G Bjorkholm M Peterson C. Hypocholesterolaemia in malignancy due to elevated low-density-lipoprotein-receptor activity in tumour cells: evidence from studies in patients with leukaemia. Lancet. (1985) 2:1150–4. 10.1016/S0140-6736(85)92679-0 2865616

[139] Scribano D Baroni S Pagano L Zuppi C Leone G Giardina B. Return to normal values of lipid pattern after effective chemotherapy in acute lymphoblastic leukemia. Haematologica. (1996) 81:343–45. 8870380

[140] Moschovi M Trimis G Apostolakou F Papassotiriou I Tzortzatou-Stathopoulou F. Serum lipid alterations in acute lymphoblastic leukemia of childhood. J Pediatr Hematol Oncol. (2004) 26:289–93. 10.1097/00043426-200405000-00006 15111780

[141] Liebes LF Pelle E Zucker-Franklin D Silber R. Comparison of lipid composition and 1,6-diphenyl-1,3,5-hexatriene fluorescence polarization measurements of hairy cells with monocytes and lymphocytes from normal subjects and patients with chronic lymphocytic leukemia. Cancer Res. (1981) 41:4050–6. 7285012

[142] Yachnin S Golomb HM West EJ Saffold C. Increased cholesterol biosynthesis in leukemic cells from patients with hairy cell leukemia. Blood. (1983) 61:50–60. 6600213

[143] Golomb HM Saffold CW Nathans AH Dawson G. Phospholipid and cholesterol differences amongst leukemic cell types with special reference to hairy cell leukemia: a preliminary report. Clin Chim Acta. (1981) 116:311–8. 10.1016/0009-8981(81)90050-4 7296894

[144] Clutterbuck RD Millar BC Powles RL Newman A Catovsky D Jarman M. Inhibitory effect of simvastatin on the proliferation of human myeloid leukaemia cells in severe combined immunodeficient (SCID) mice. Br J Haematol. (1998) 102:522–7. 10.1046/j.1365-2141.1998.00783.x 9695968

[145] Dimitroulakos J Nohynek D Backway KL Hedley DW Yeger H Freedman MH. Increased sensitivity of acute myeloid leukemias to lovastatin-induced apoptosis: a potential therapeutic approach. Blood. (1999) 93:1308–18. 9949174

[146] Dimitroulakos J Thai S Wasfy GH Hedley DW Minden MD Penn LZ. Lovastatin induces a pronounced differentiation response in acute myeloid leukemias. Leuk Lymphoma. (2000) 40:167–78. 10.3109/10428190009054894 11426618

[147] Perez-Sala D Mollinedo F. Inhibition of isoprenoid biosynthesis induces apoptosis in human promyelocytic HL-60 cells. Biochem Biophys Res Commun. (1994) 199:1209–15. 10.1006/bbrc.1994.1359 8147861

[148] Wang IK Lin-Shiau SY Lin JK. Induction of apoptosis by lovastatin through activation of caspase-3 and DNase II in leukaemia HL-60 cells. Pharmacol Toxicol. (2000) 86:83–91. 10.1034/j.1600-0773.2000.d01-16.x 10728920

[149] Lishner M Bar-Sef A Elis A Fabian I. Effect of simvastatin alone and in combination with cytosine arabinoside on the proliferation of myeloid leukemia cell lines. J Investig Med. (2001) 49:319–24. 10.2310/6650.2001.33896 11478407

[150] Wong WW Tan MM Xia Z Dimitroulakos J Minden MD Penn LZ. Cerivastatin triggers tumor-specific apoptosis with higher efficacy than lovastatin. Clin Cancer Res. (2001) 7:2067–75. 11448925

[151] Holstein SA Hohl RJ. Synergistic interaction of lovastatin and paclitaxel in human cancer cells. Mol Cancer Ther. (2001) 1:141–9. 12467231

[152] Li HY Appelbaum FR Willman CL Zager RA Banker DE. Cholesterol-modulating agents kill acute myeloid leukemia cells and sensitize them to therapeutics by blocking adaptive cholesterol responses. Blood. (2003) 101:3628–34. 10.1182/blood-2002-07-2283 12506040

[153] Maksumova L Ohnishi K Muratkhodjaev F Zhang W Pan L Takeshita A. Increased sensitivity of multidrug-resistant myeloid leukemia cell lines to lovastatin. Leukemia. (2000) 14:1444–50. 10.1038/sj.leu.2401856 10942241

[154] Newman A Clutterbuck RD Powles RL Millar JL. Selective inhibition of primary acute myeloid leukaemia cell growth by simvastatin. Leukemia. (1994) 8:2023–9. 7967748

[155] Sassano A Katsoulidis E Antico G Altman JK Redig AJ Minucci S. Suppressive effects of statins on acute promyelocytic leukemia cells. Cancer Res. (2007) 67:4524–32. 10.1158/0008-5472.CAN-06-3686 17483369

[156] Stirewalt DL Appelbaum FR Willman CL Zager RA Banker DE Mevastatin can increase toxicity in primary AMLs exposed to standard therapeutic agents, but statin efficacy is not simply associated with ras hotspot mutations or overexpression. Leuk Res. (2003) 27:133–45. 10.1016/S0145-2126(02)00085-1 12526919

[157] Xia Z Tan MM Wong WW Dimitroulakos J Minden MD Penn LZ. Blocking protein geranylgeranylation is essential for lovastatin-induced apoptosis of human acute myeloid leukemia cells. Leukemia. (2001) 15:1398–407. 10.1038/sj.leu.2402196 11516100

[158] Wu J Wong WW Khosravi F Minden MD Penn LZ. Blocking the Raf/MEK/ERK pathway sensitizes acute myelogenous leukemia cells to lovastatin-induced apoptosis. Cancer Res. (2004) 64:6461–8. 10.1158/0008-5472.CAN-04-0866 15374955

[159] Chen R Xiao W Li D Mu S. Combination of simvastatin and imatinib sensitizes the CD34+ cells in K562 to cell death. Med Oncol. (2011) 28:528–31. 10.1007/s12032-010-9472-9 20354828

[160] Yang YC Xiao DW Liu H Chuan LM Zeng YL Zhou DA Liu W Xu GQ Huang WF. Mechanism of simvastatin-induced K562 cell apoptosis. Pharmacology. (2009) 84:191–5. 10.1159/000235907 19729986

[161] Oh B Kim TY Min HJ Kim M Kang MS Huh JY Kim Y Lee DS. Synergistic killing effect of imatinib and simvastatin on imatinib-resistant chronic myelogenous leukemia cells. Anticancer Drugs. (2013) 24:20–31. 10.1097/CAD.0b013e32835a0fbd 23075630

[162] Griner LN McGraw KL Johnson JO List AF Reuther GW. JAK2-V617F-mediated signalling is dependent on lipid rafts and statins inhibit JAK2-V617F-dependent cell growth. Br J Haematol. (2013) 160:177–87. 10.1111/bjh.12103 23157224 PMC4505927

[163] Yasuda N Matzno S Iwano C Nishikata M Matsuyama K. Evaluation of apoptosis and necrosis induced by statins using fluorescence-enhanced flow cytometry. J Pharm Biomed Anal. (2005) 39:712–7. 10.1016/j.jpba.2005.04.022 15927433

[164] Sheen C Vincent T Barrett D Horwitz EM Hulitt J Strong E Grupp SA Teachey DT. Statins are active in acute lymphoblastic leukaemia (ALL): a therapy that may treat ALL and prevent avascular necrosis. Br J Haematol. (2011) 155:403–7. 10.1111/j.1365-2141.2011.08696.x 21554258 PMC4119812

[165] Chapman-Shimshoni D Yuklea M Radnay J Shapiro H Lishner M. Simvastatin induces apoptosis of B-CLL cells by activation of mitochondrial caspase 9. Exp Hematol. (2003) 31:779–83. 10.1016/S0301-472X(03)00192-9 12962723

[166] Podhorecka M Halicka D Klimek P Kowal M Chocholska S Dmoszynska A. Simvastatin and purine analogs have a synergic effect on apoptosis of chronic lymphocytic leukemia cells. Ann Hematol. (2010) 89:1115–24. 10.1007/s00277-010-0988-z 20499237 PMC2940031

[167] Vitols S Angelin B Juliusson G. Simvastatin impairs mitogen-induced proliferation of malignant B-lymphocytes from humans–*in vitro* and *in vivo* studies. Lipids. (1997) 32:255–62. 10.1007/s11745-997-0032-1 9076662

[168] Nonaka M Uota S Saitoh Y Takahashi M Sugimoto H Amet T. Role for protein geranylgeranylation in adult T-cell leukemia cell survival. Exp Cell Res. (2009) 315:141–50. 10.1016/j.yexcr.2008.10.010 18992741

[169] Rozados VR Hinrichsen LI Binda MM Gervasoni SI Matar P Bonfil RD. Lovastatin enhances the antitumoral and apoptotic activity of doxorubicin in murine tumor models. Oncol Rep. (2008) 19:1205–11. 10.3892/or.19.5.1205 18425377

[170] van de Donk NW Schotte D Kamphuis MM van Marion AM van Kessel B Bloem AC. Protein geranylgeranylation is critical for the regulation of survival and proliferation of lymphoma tumor cells. Clin Cancer Res. (2003) 9:5735–48. 14654559

[171] Cafforio P Dammacco F Gernone A Silvestris F. Statins activate the mitochondrial pathway of apoptosis in human lymphoblasts and myeloma cells. Carcinogenesis. (2005) 26:883–91. 10.1093/carcin/bgi036 15705602

[172] Dmoszynska A Podhorecka M Klimek P Grzasko N. Lovastatin and thalidomide have a combined effect on the rate of multiple myeloma cell apoptosis in short term cell cultures. Eur J Clin Pharmacol. (2006) 62:325–9. 10.1007/s00228-006-0106-2 16523333

[173] Gronich N Drucker L Shapiro H Radnay J Yarkoni S Lishner M. Simvastatin induces death of multiple myeloma cell lines. J Investig Med. (2004) 52:335–44. 10.1136/jim-52-05-34 15551657

[174] Hus M Grzasko N Szostek M Pluta A Helbig G Woszczyk D. Thalidomide, dexamethasone and lovastatin with autologous stem cell transplantation as a salvage immunomodulatory therapy in patients with relapsed and refractory multiple myeloma. Ann Hematol. (2011) 90:1161–6. 10.1007/s00277-011-1276-2 21698395 PMC3168480

[175] van der Spek E Bloem AC Lokhorst HM van Kessel B Bogers-Boer L van de Donk NW. Inhibition of the mevalonate pathway potentiates the effects of lenalidomide in myeloma. Leuk Res. (2009) 33:100–8. 10.1016/j.leukres.2008.06.001 18621417

[176] van de Donk NW Kamphuis MM van Kessel B Lokhorst HM Bloem AC. Inhibition of protein geranylgeranylation induces apoptosis in myeloma plasma cells by reducing Mcl-1 protein levels. Blood. (2003) 102:3354–62. 10.1182/blood-2003-03-0970 12855556

[177] Hartwell KA Miller PG Mukherjee S Kahn AR Stewart AL Logan DJ. Niche-based screening identifies small-molecule inhibitors of leukemia stem cells. Nat Chem Biol. (2013) 9:840–8. 10.1038/nchembio.1367 24161946 PMC4009363

[178] Christ M Luu B Mejia JE Moosbrugger I Bischoff P. Apoptosis induced by oxysterols in murine lymphoma cells and in normal thymocytes. Immunology. (1993) 78:455–60. 7682990 PMC1421833

[179] Hwang PL. Inhibitors of protein and RNA synthesis block the cytotoxic effects of oxygenated sterols. Biochim Biophys Acta. (1992) 1136:5–11. 10.1016/0167-4889(92)90077-O 1379472

[180] Hietter H Bischoff P Beck JP Ourisson G Luu B. Comparative effects of 7 beta-hydroxycholesterol towards murine lymphomas, lymphoblasts and lymphocytes: selective cytotoxicity and blastogenesis inhibition. Cancer Biochem Biophys. (1986) 9:75–83. 3815322

[181] Rosa Fernandes L Stern AC Cavaglieri RC Nogueira FC Domont G. 7-Ketocholesterol overcomes drug resistance in chronic myeloid leukemia cell lines beyond MDR1 mechanism. J Proteomics. (2017) 151:12–23. 10.1016/j.jprot.2016.06.011 27343758

[182] Aupeix K Weltin D Mejia JE Christ M Marchal J Freyssinet JM. Oxysterol-induced apoptosis in human monocytic cell lines. Immunobiology. (1995) 194:415–28. 10.1016/S0171-2985(11)80108-7 8749234

[183] Lim HK Kang HK Yoo ES Kim BJ Kim YW Cho M Oxysterols induce apoptosis and accumulation of cell cycle at G/M phase in the human monocytic THP-1 cell line. Life Sci. (2003) 72:1389–99. 10.1016/S0024-3205(02)02377-9 12527036

[184] Tsujioka T Yokoi A Itano Y Takahashi K Ouchida M Okamoto S. Five-aza-2′-deoxycytidine-induced hypomethylation of cholesterol 25-hydroxylase gene is responsible for cell death of myelodysplasia/leukemia cells. Sci Rep. (2015) 5:16709. 10.1038/srep16709 26577244 PMC4649363

[185] Bonig H Papayannopoulou T. Hematopoietic stem cell mobilization: updated conceptual renditions. Leukemia. (2013) 27:24–31. 10.1038/leu.2012.254 22951944 PMC3676423

[186] Giralt S Costa L Schriber J Dipersio J Maziarz R McCarty J. Optimizing autologous stem cell mobilization strategies to improve patient outcomes: consensus guidelines and recommendations. Biol Blood Marrow Transplant. (2014) 20:295–308. 10.1016/j.bbmt.2013.10.013 24141007

[187] Tabas I Lichtman AH. Monocyte-macrophages and T cells in atherosclerosis. Immunity. (2017) 47:621–34. 10.1016/j.immuni.2017.09.008 29045897 PMC5747297

[188] Nahrendorf M. Myeloid cell contributions to cardiovascular health and disease. Nat Med. (2018) 24:711–20. 10.1038/s41591-018-0064-0 29867229 PMC7301893

[189] Zmyslowski A Szterk A. Current knowledge on the mechanism of atherosclerosis and pro-atherosclerotic properties of oxysterols. Lipids Health Dis. (2017) 16:188. 10.1186/s12944-017-0579-2 28969682 PMC5625595

[190] Nofer JR. Estrogens and atherosclerosis: insights from animal models and cell systems. J Mol Endocrinol. (2012) 48:R13–29. 10.1530/JME-11-0145 22355098

[191] Dutta P Courties G Wei Y Leuschner F Gorbatov R Robbins CS. Myocardial infarction accelerates atherosclerosis. Nature. (2012) 487:325–9. 10.1038/nature11260 22763456 PMC3401326

[192] Dutta P Sager HB Stengel KR Naxerova K Courties G Saez B. Myocardial infarction activates CCR2(+) hematopoietic stem and progenitor cells. Cell Stem Cell. (2015) 16:477–87. 10.1016/j.stem.2015.04.008 25957903 PMC4426344

